# Motor network dynamic resting state fMRI connectivity of neurotypical children in regions affected by cerebral palsy

**DOI:** 10.3389/fnhum.2024.1339324

**Published:** 2024-05-21

**Authors:** Varina L. Boerwinkle, Bethany L. Sussman, Laura de Lima Xavier, Sarah N. Wyckoff, William Reuther, Michael C. Kruer, Martin Arhin, Justin M. Fine

**Affiliations:** ^1^Division of Pediatric Neurology, University of North Carolina at Chapel Hill, Chapel Hill, NC, United States; ^2^Division of Neurosciences, Barrow Neurological Institute at Phoenix Children’s Hospital, Phoenix, AZ, United States; ^3^Division of Neonatology, Center for Fetal and Neonatal Medicine, Children’s Hospital Los Angeles, Los Angeles, CA, United States; ^4^Brainbox Inc., Baltimore, MD, United States; ^5^Departments of Child Health, Neurology, Genetics and Cellular & Molecular Medicine, University of Arizona College of Medicine – Phoenix, Phoenix, AZ, United States; ^6^Department of Neurosurgery, Baylor College of Medicine, Houston, TX, United States

**Keywords:** effective connectivity, cerebral palsy, resting state functional MRI, normative, motor network connectivity

## Abstract

**Background:**

Normative childhood motor network resting-state fMRI effective connectivity is undefined, yet necessary for translatable dynamic resting-state-network-informed evaluation in pediatric cerebral palsy.

**Methods:**

Cross-spectral dynamic causal modeling of resting-state-fMRI was investigated in 50 neurotypically developing 5- to 13-year-old children. Fully connected six-node network models per hemisphere included primary motor cortex, striatum, subthalamic nucleus, globus pallidus internus, thalamus, and contralateral cerebellum. Parametric Empirical Bayes with exhaustive Bayesian model reduction and Bayesian modeling averaging informed the model; Purdue Pegboard Test scores of hand motor behavior were the covariate at the group level to determine the effective-connectivity-functional behavior relationship.

**Results:**

Although both hemispheres exhibited similar effective connectivity of motor cortico-basal ganglia-cerebellar networks, magnitudes were slightly greater on the right, except for left-sided connections of the striatum which were more numerous and of opposite polarity. Inter-nodal motor network effective connectivity remained consistent and robust across subjects. Age had a greater impact on connections to the contralateral cerebellum, bilaterally. Motor behavior, however, affected different connections in each hemisphere, exerting a more prominent effect on the left modulatory connections to the subthalamic nucleus, contralateral cerebellum, primary motor cortex, and thalamus.

**Discussion:**

This study revealed a consistent pattern of directed resting-state effective connectivity in healthy children aged 5–13 years within the motor network, encompassing cortical, subcortical, and cerebellar regions, correlated with motor skill proficiency. Both hemispheres exhibited similar effective connectivity within motor cortico-basal ganglia-cerebellar networks reflecting inter-nodal signal direction predicted by other modalities, mainly differing from task-dependent studies due to network differences at rest. Notably, age-related changes were more pronounced in connections to the contralateral cerebellum. Conversely, motor behavior distinctly impacted connections in each hemisphere, emphasizing its role in modulating left sided connections to the subthalamic nucleus, contralateral cerebellum, primary motor cortex, and thalamus. Motor network effective connectivity was correlated with motor behavior, validating its physiological significance. This study is the first to evaluate a normative effective connectivity model for the pediatric motor network using resting-state functional MRI correlating with behavior and serves as a foundation for identifying abnormal findings and optimizing targeted interventions like deep brain stimulation, potentially influencing future therapeutic approaches for children with movement disorders.

## Highlights

- This is the first normative model of effective connectivity of motor networks in children at rest, paving the way for allowing comparisons with children who have motor network pathology.
- A fully connected model of effective connectivity of motor networks was estimated for each hemisphere in children 5- to 13-year-old children, and the connections were covaried with age and motor skill.
- While the motor network effective connectivity between hemispheres was similar for age-associated changes, contrastingly those for motor-skill differed.
- Thus, age or developmental impact on the motor network as a more generalized pattern across hemispheres effective connectivity, whereas motor-skill has asymmetrical influences.

## Introduction

Medication-based therapy for cerebral palsy (CP), which impacts 1.5 to 2.5 per 1,000 live births, is often unsatisfactory (Elia et al., 2018). One promising solution for those with symptoms of athetosis, chorea or dystonia, which are found in many subtypes of CP, is deep brain stimulation (DBS), which is limited by lack of precision-based models to direct the DBS lead placement with less than 50–75% success, and frequent complications (Elia et al., 2018; Koy et al., 2023). Comparatively, in diseases such as Parkinson’s disease, DBS lead placement informed by rs-fMRI analysis of the *motor networks (MN)*, has improved outcomes (Horn et al., 2017). To create such precision-based biomarkers for DBS lead placement for CP from non-invasive modalities, as compared to other invasive and higher risk methods such as stereo-electroencephalogram (Sanger et al., 2018), such as resting state fMRI (rs-fMRI), first normative age-related characterization must occur, which is the focus of this study.

Much is known about the motor networks neuroanatomical function, which involves parallel-interdependent loops, creating circuit redundancy and pathology-resistance (Alexander et al., 1986; Haber, 2003). These pathways are also reflected in rs-fMRI networks (RSN) *and are aligned with motor behavior across the lifespan from childhood into adulthood* (Allen et al., 2011; Manza et al., 2015; Solé-Padullés et al., 2016). For instance, regions of the basal ganglia (BG) network exhibit childhood-age-dependent FC increases that are associated with adult age-related changes, including connectivity with cortical regions (Solé-Padullés et al., 2016). Further, some portions of the MN FC in children show age-and-location-dependent laterality shifts, resulting in overall less lateralization with age, whereas even some MN networks do not shift in lateralization with age (Agcaoglu et al., 2021). Given these findings, we anticipated age-related MN connectivity changes, and a relative degree of hemispheric symmetry of MN in children, with potential asymmetry of connections involving the striatum.

Further, considering the two primary options of rs-fMRI analysis, which are *effective connectivity (EC)*, meaning informing on the direction of signal between location, and *functional connectivity (FC)*, meaning static relationship between locations—have had differences in the impact on DBS-motor therapy, with the former being more impactful (Sussman et al., 2022). In our recent literature review comparing the utility of FC and EC in MN impairments in children and adult, it appears that, for movement disorders, EC may have unique information that could be particularly useful in neuromodulation (Sussman et al., 2022). Unlike static FC, which relies on temporal correlations in fMRI activity, EC incorporates time-dependent causal signaling between network locations, called nodes. EC quantifies the nodal-directed connectivity magnitude and polarity (i.e., excitatory, or inhibitory), inferring a causal relationship. For example, EC in essential tremor (ET) detects different EC during tremor among MN nodes, even at rest (Park et al., 2017), as reviewed concerning age in Sussman et al. (2022). Accordingly, EC predicts surgical outcomes in adults with essential tremor (Park et al., 2017) and Parkinson’s disease in adults (Kahan et al., 2014, 2019). Thus, evaluation through EC-hypothesis-driven nodal relationships and network configurations allows the determination of which model, if any, explains the system signal variance best and locates the sources and sinks of the atypical signal.

However, MN normative pediatric EC is not as well understood as FC. EC leverages temporal or spectral aspects of rs-fMRI activity in multiple regions to model the directional causality of activity between regions (Friston et al., 2003, 2014). EC and FC relationships can, but do not necessarily, follow direct anatomical connections and do not negate the possibility of other anatomical connections influencing connectivity. A well-established method of modeling EC is dynamic causal modeling (DCM). DCM is a model-based approach where excitatory versus inhibitory influences between brain regions are modeled, and competing models with the same nodes (but different connections) are compared for best fit using Bayesian estimation and comparison methods (Penny et al., 2006; Friston et al., 2015).

Importantly, DCM does not assume direct anatomical connectivity between nodes in a model. Modulatory influences from one node to another indicate that an increase in activity in one node caused an increase or decrease in activity in the other, rather than direct monosynaptic activity. The causal relationship in EC methods such as DCM contrasts with FC, which is measured as correlated static activity within and across brain regions, without causation. FC also does not make assumptions about structural (synaptic) connections between regions (Friston et al., 2003, 2014).

However, most investigations of MN EC only capture volitional activity, which is less feasible than resting-state studies in children due to their lower ability to cooperate (Fassbender et al., 2017; Sussman et al., 2022). For instance, a study on motor-sequence learning in adults revealed that early encoding of motor-sequence learning was associated with negative modulation of several connections between the primary motor cortex (M1), cerebellum, premotor cortex, and the putamen. However, only negative modulation from the left cerebellum (CER) to right putamen was associated with motor-sequence learning after memory consolidation (Tzvi et al., 2015). When comparing active-task and resting state in the same individuals, it is largely unknown which has greater implications for improving DBS outcomes (Sussman et al., 2022). Though there are expected active vs. resting-state-based differences in connectivity, the key factor is aggregating enough data to determine which, if any connection, from either state, is associated with DBS outcomes targeting the same location. However, such volitional studies are not a feasible methodological choice in children with CP, who move frequently and involuntarily, and scanning conditions require the patient to be perfectly still, awake, and cooperative for relatively extended periods of time.

Hence the primary aim of this study was to create a prototypical resting-state MN EC model in typically developing young children, for later comparison of the same study in children with CP, encompassing cortical and subcortical MN circuitry, which are the primary regions impacted in cerebral palsy (Kelly et al., 2015; Lazzari et al., 2015; Elia et al., 2018; Wolf et al., 2019; Sadowska et al., 2020; Koy et al., 2023). Thus, the study sought to: (1) identify the effective dynamics of the MN circuitry in children at rest—i.e., while not initiating, stopping, or ‘tuning’ a motor action—and not time-locked to movement events (e.g., tremors), and (2) evaluate these connections for association with age and motor skill. Based on previous research, we anticipated EC to be predominantly symmetric across hemispheres, with the strength of connections between regions correlated with motor skills.

Investigating EC enables inferences about directional connectivity between locations, which may have future implications for the treatment of movement disorders in children. The purpose of this study is to develop an EC model for comparative purposes rather than to differentiate models in a clinical vs. non-clinical groups. Since the resulting models from DCM are influenced by factors such as participant selection, nodes, and connections, we opted to use a cohort of typically developing children (to facilitate broader comparison) and employ data-driven model reduction methods after selecting nodes.

## Methods

The Institutional Review Board (IRB) of Phoenix Children’s Hospital (PCH) and National Institutes of Mental Health (NIMH) approved Cincinnati MR Imaging of Neurodevelopment Database (C-MIND; https://cmind.research.cchmc.org/; see acknowledgments) data access, which were acquired with informed consent. Both the initial data acquisition and current secondary analysis were completed by the Declaration of Helsinki’s ethical principles for medical research involving human subjects. The imaging and behavioral data used for this study are available through the NIMH Data Archive, through a data use agreement between the NIMH Data Archive and qualifying institutions. The metadata for this study are available in Supplementary Table S1 and through this study’s entry in the NIMH Data Archive.

### Inclusion and exclusion criteria

Seventy participants were selected from a national functional neuroimaging and behavioral database from typically developing children released by the Pediatric Functional Neuroimaging Research Network. The Cincinnati MR Imaging of NeuroDevelopment (C-MIND) database is publicly accessible online at http://research.cchmc.org/c-mind. To ensure that the C-MIND database contains data from normally developing children, the following original inclusion criteria were applied, as reported in similar prior studies (Vannest et al., 2014), which were: (1) negative history and family history (in first-degree relatives) of neurological or psychiatric disease; (2) Body Mass Index between the 5 and 95th percentile for age and gender and; (3) normal neurological exam. Exclusion criteria included: (1) chronic illness; (2) gestation less than 37 weeks or greater than 42 weeks; (3) birth weight less than the 10th percentile; (4) history of head trauma with a loss of consciousness; (5) special education; (6) orthodontic braces or other metallic implants, and (7) standard MRI contraindications. In this original study, informed consent was obtained from the parent or guardian, per the consent procedures were approved by the Institutional Review Board of the Cincinnati Children’s Hospital Medical Center. The current study further selected for exclusion of left-handedness, age less than 5 or above 13 years of age at the time of the rs-fMRI, greater than 3 mm of head movement in any direction during the rs-fMRI, lack of T1W MRI, lack of Purdue Pegboard Test (PPBT) results for both the left- and right-hand function. Since some participants in this study had up to three visits, the data from the first visit where a participant matched all these criteria was used. Additional visits for the participants, if any, were not used. Of the 70 subjects’ data evaluated, 50 met the study criteria.

#### Purdue pegboard test

The PPBT measures manual dexterity and gross motor coordination of the arms and hands (Tiffin and Asher, 1948). The available data for the PPBT scores, which was the only motor skill test acquired in the research data base with rs-fMRI in normally developing children, were raw scores for the dominant hand, non-dominant hand, and ‘both hand’ tasks. Since PPBT is expected to correlate with age, these scores were converted to an age and sex-based standardized form. Raw scores for the dominant and non-dominant hand were recoded to reflect how many standard deviations (SD) they were from the mean score for a child for that age (at 6-month intervals) and gender based on the Lafayette User Manual (Podell, 2011). Thus, scores within the first SD were coded as 0, and scores that were more than one SD from the mean were coded to reflect the number and direction of SD they were from the mean (e.g., 1, 2, 3…, −1, −2, −3, etc.).

The participants’ converted PPBT scores as a number of standard deviations are provided in Supplementary Table S1. This table also details each connection for each participant (Podell, 2011). Per the data sharing agreement with the NIH NDA, we are not able to provide each participant’s raw scores nor individual age or other such scores taken directly from the data repository.

#### Acquisition and preprocessing

A Philips 3 T Achieva MRI, the 32-channel head coil was used to acquire a T1W image and a five-minute (150 volume) fMRI scan. Scan Parameters: (1) T1W—standard inversion recovery prepared method, repetition time (TR) 8.1 msec, echo time (TE) 3.7 msec, flip angle (FA) 8°, shot interval 2,800 msec, inversion time (TI) 939 msec, turbo field echo (TFE) shot duration/acquisition, 1851.9/1804.6 msec, field of view (FOV) 256 × 224 × 160 mm, voxel size 1 × 1 × 1 mm acquired in the sagittal plane with sensitivity encoding (SENSE) 5 min 15 s. (2) rs-fMRI—TR 2,000 msec, TE 35 msec, FA 90°, FOV 240 × 240 × 144 mm, matrix size 80 × 80, slice thickness of 4 mm, voxel size 3 × 3 × 4 mm, and 36 transverse slices of the whole brain. A standard rs-fMRI preprocessing pipeline (SPM12, www.fil.ion.ucl.ac.uk/spm, Wellcome Trust Centre for Neuroimaging, London, United Kingdom) (Friston et al., 1994) was utilized to eliminate the first 5 volumes, slice-timing correction then realignment, and linear coregistration of the echo planar imaging (EPI) data to the T1W, with no spatial smoothing. Spatially smoothed data were not used for the functional data entered into main dynamic causal modeling (DCM) analyses to increase the precision of regions of interest (ROI) and avoid artificially affecting connectivity between the ROIs, especially given the differences in size across ROIs and variability of proximity between the ROIs (Alakörkkö et al., 2017). SPM12 and CAT12 (Gaser and Dahnke, 2016) were used to segment the T1W, which were non-linearly registered to MNI space. CAT12 was then used to deform the relevant motor-related atlases [HMAT (Mayka et al., 2006); BGHAT (Prodoehl et al., 2008); and IBSR template (Filipek et al., 1994)] to subject space for ROI specification.

### Denoising

Before running a GLM, and after motion correction realignment the rs-fMRI datasets that passed initial quality control checks (i.e., <3 mm movement in any direction) were denoised using the ICA-based Automatic Removal Of Motion Artifacts (ICA-AROMA) (Pruim et al., 2015) program. Since ICA-AROMA is designed for smoothed data in MNI space with an FMRIB Software Library (FSL) bounding box, a copy of the subject-space data was first smoothed with an 8 mm FWHM Gaussian kernel in SPM. FSL’s FLIRT (Jenkinson and Smith, 2001; Jenkinson et al., 2002; Greve and Fischl, 2009) was then used to co-register the BOLD data to the T1 and FSL’s FNIRT (Smith et al., 2004; Andersson et al., 2010; Jenkinson et al., 2012) was used to normalize to the MNI 152 template. Since it was planned to use unsmoothed data in the DCM, the MELODIC (Smith et al., 2009) portion of ICA-AROMA performed on the participants’ smoothed data, and then the MELODIC results from the smoothed data were fed into ICA-AROMA and non-aggressive denoising was performed on the unsmoothed data.

### General linear model

After ICA-AROMA denoising, a GLM was first estimated with the denoised data over the whole brain to extract nuisance regressor time series from the cerebrospinal fluid (CSF) mask calculated in CAT12. A GLM was then re-estimated from the functional data with a high-pass filter with a cut-off frequency of 1/128 s to remove low-frequency non-neural artifacts from data and included the extra-cerebral time-series nuisance regressor and six nuisance regressors that captured head motion (Friston et al., 2003). Low-pass filtering (e.g., > 0.1 Hz) was not used because data in those frequencies may contain meaningful information in rs-fMRI studies (Chen and Glover, 2015; Lin et al., 2015) and may be informative for comparison with clinical populations (Boerwinkle et al., 2017). Time courses from the results this second GLM were then extracted from the data and used for dynamic causal modeling as detailed below.

#### EC analysis

The models were inverted using bilinear cross-spectral DCM using DCM 12.5 (Friston et al., 2003, 2014) and detailed below.

#### ROI selection

**Figure 1:**
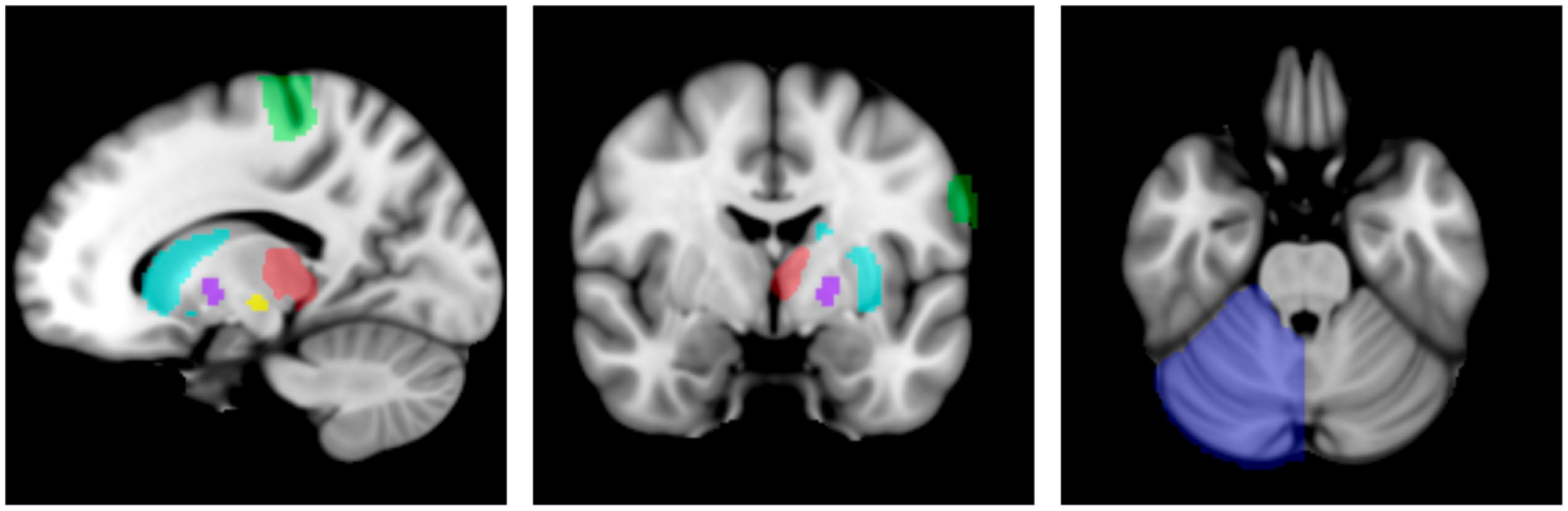
Regions of interest. Sagittal, coronal, and axial views of the regions of interest in the left hemisphere model (radiological view). The right hemisphere model contains the same regions of interest with the opposite lateralization of each ROI. Key: Primary motor cortex (M1): green, striatum: cyan, subthalamic nucleus (STN): yellow, globus pallidus internus (GPi): purple, thalamus: red, contralateral cerebellum: blue. Atlases used: HMAT: M1; BGHAT: striatum (combination of caudate and putamen ROIs), STN, GPi; IBSR: thalamus, cerebellum. This figure is represented in MNI space, however, for analysis, ROIs were projected into subject anatomical space.

Six MN ROI per hemisphere were selected to incorporate key locations in the cortico-striato-pallido-thalamo-cortical and cerebello-thalamo-cortical pathways (Haber, 2003; Milardi et al., 2019; Quartarone et al., 2019) including the primary motor cortex (M1), striatum (STR), globus pallidus internus (GPi), subthalamic nucleus (STN), thalamus (THAL), and the contralateral cerebellum (CER). Each ROI location was identified from the predefined referenced atlases. Thus, a six-ROI (node) model was specified per hemisphere per subject (Figure 1). Hemispheres were modeled separately to reduce the complexity of the models.

#### ROI data extraction and preprocessing

The first eigenvariate of the rs-fMRI signal from the preprocessed and GLM-estimated six nodes were extracted. The first principal component eigenvariate was selected as it represented the dominant variation of activity in that node. Steady-state spectral amplitude and phase representations of each node’s activity were then obtained through the Fourier transform. The local spectrum of each region was modeled as a power law distribution with an amplitude and scale, the latter indicating the frequency by amplitude slope (Friston et al., 2014). Thus, directional connectivity by DCM is achieved through EC estimated parameters of nodal frequency, auto- and cross-spectrum (herein also termed EC), between regions through multivariate auto-regression models of rs-fMRI blood oxygen derived (BOLD) signal.

#### Primary-level DCM

Variational Bayesian inversion was used to fit the differential equation connectivity model (Penny et al., 2010, 2011). This method of inversion involves fitting the DCM to maximize the likelihood of the model under prior EC parameter specifications. Model reduction or selection was not performed at the first level as this is the individual level. Instead, we estimated models at the first level using an iterative empirical Bayes inversion scheme that alternated between estimating individual DCMs and estimating group effects to use as DCM priors. We chose to do this because this method reduces the effects of local optima and draws subjects toward the group mean (Friston et al., 2015; Zeidman et al., 2019).

##### Group level modeling

Parametric Empirical Bayes (PEB) second-level DCM was then performed (Friston et al., 2015; Zeidman et al., 2019). PEB was used to create a group average for each hemisphere that preserved commonalities across the group *and differences associated with age and motor skills*. In addition to the group average, for each model, the mean-centered age at the time of scan and SD-coded PPBT from the contralateral hand were entered as covariates. This was done to determine if any EC parameters were associated with age-related motor skill, herein termed *EC-age and EC-PPBT, respectively to denote the brain-age and brain-behavior associations.* Since age and motor skill are likely related, we computed the variance inflation factor (VIF) between age and PPBT for each hemisphere using a maximum VIF of 2.5 as our threshold for avoiding multicollinearity in the PEB analysis (Mansfield and Helms, 1982).

### Bayesian model network thresholding

Because the goal was to create a proto-typical model of connectivity, it was assumed that all subjects would have the same optimal model structure but may differ in EC parameter estimates. To maximize the similarities between subjects, a parametric empirical Bayes framework was used for the group. PEB places greater weight on subjects and EC parameters that are less ‘noisy’ (Friston et al., 2015). Next, Bayesian Model Comparison (BMC) through exhaustive Bayesian Model Reduction (BMR) (Friston et al., 2016) was performed on the full model space of each group hemisphere model, separately. Bayesian Model Reduction is a Bayesian Model Comparison method that effectively “prunes” redundant or uninformative EC parameters by using a greedy search for switching EC parameters and combinations of EC parameters of a fully connected and estimated model “on” and “off” and then efficiently computing the evidence and probability of each potential reduced model. Bayesian Model Reduction also favors the simplest model that can explain the data (Friston et al., 2016). Bayesian Model Reduction was chosen over selecting specific models to compare and utilize the more data-driven model selection method in an essentially hypothesis-driven paradigm.

Additionally, the investigators chose not to impose restrictions on connections that represent the direct, indirect, and hyperdirect pathways during model comparisons. The primary reason for not imposing such restrictions was the prior information about these pathways is based on active paradigms (movement initiation, cessation, inhibition, etc.) rather than an extended period of rest. Additionally, restricting connections to known anatomical connections and pathways also risks preventing the detection of indirect modulatory effects. For example, while there is no known THAL➔CER anatomical connection, thalamic DBS has been shown to increase contralateral cerebellar activity (Gibson et al., 2016) and thalamotomy has been associated with increases in THAL➔CER EC (Park et al., 2017). This suggests that it is still prudent to query functional connections without a known direct anatomical connection, because they may have an indirect connection relevant to motor function, pathology, and therapy.

Through Bayesian Model Reduction, the similarities across the group were evaluated with a greedy search of exhaustive potential models and provided with the optimized explanatory model. Thus, a Bayesian Model Average (BMA) was calculated over the 256 best models from the BMR to obtain the optimized model EC parameters by weighing their posterior probability (Penny et al., 2006; Friston et al., 2015). Further discussion is limited to EC parameters with posterior parameter estimates with evidence past the threshold of >95% free energy modeled.

To determine the effect sizes and whether there is a predictive ability of the *EC-age* and *EC-PPBT* correlations, we used leave-one-out cross-validation tests (LOO-CV). The methods for selecting (multiple) parameters to include for LOO-CV are relatively new, but a proposed technique when there are multiple posterior (covariate) parameters with evidence is to select those with the highest effect sizes to include in the LOO-CV test (including the entire A matrix may dilute any interesting effects). Therefore, we performed LOO-CV tests by calculating one test per hemisphere-covariate combination (four tests total). For each respective hemisphere covariate combination, the LOO-CV test included all the parameters that had >0.95 posterior probability in the PEB.

### Hemisphere differences

To investigate hemispheric symmetry and identify statistically significant interhemispheric differences, we also constructed a hierarchical PEB (PEB of PEBs) where the separate hemisphere PEBs were entered in the order of right then left. In the design matrix, the right PEB was coded as −1 and the left as 1. Bayesian Model Reduction and Bayesian Model Averaging were also performed as above. To avoid bias toward our hypothesis of hemispheric symmetry, the discussion of EC parameter similarity between hemispheres is limited to posterior parameter estimates with evidence past the threshold of >95% free energy, but we used a lower threshold of 75% for the evaluation of differences.

## Results

The median age for the 50 subjects (27 female) included was 9.17 years [IQR: 7.6–11.3]. The median right-hand PPBT score was 11 [IQR: 10–12.75] and the median left-hand PPBT score was 10 [IQR: 9–12] (used for correlation to contralateral hemisphere). In one subject, the right STN activity could not be extracted due to the small volume, and this subject was excluded from the right hemisphere model. Thus, 49 and 50 subjects’ data for the right and left hemisphere models, respectively, were utilized. Pearson correlation tests between age and the PPBT for the left hand (right hemisphere) was *r* = 0.75, *p* < 0.001 and for the right hand (left hemisphere) was *r* = 0.62, *p* < 0.001, suggesting likely collinearity between the predictor variables, particularly in the right hemisphere, thus justifying the use of SD-coded PPBT scores in the PEB analysis.

To describe the EC results an ‘excitatory’ connection or modulation from one location to another indicates that an increase in rs-fMRI activity in the first location causes an increase in activity in the second location. In contrast ‘inhibitory’ connections or modulations indicate that an increase in rs-fMRI activity in the first location causes a decrease in rs-fMRI activity in the second location. Importantly, this does not imply or state that there are direct structural or monosynaptic connections between the regions, or that the connectivity modeled is a direct structural connection (Jenkinson et al., 2002; Friston et al., 2003).

### Baseline connectivity

**Figure 2:**
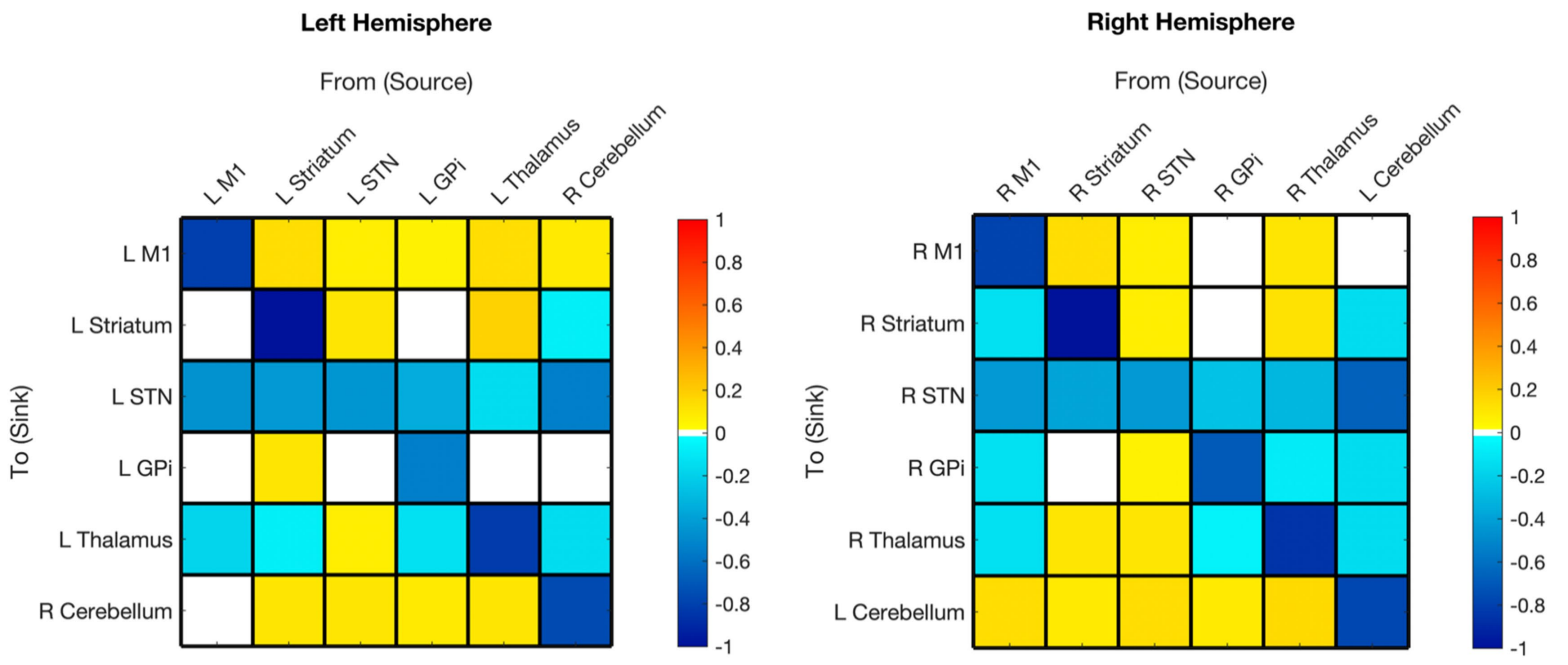
Hemispheric mean group effective connectivity. The y-axis lists the receiving nodes of the signal coming from the x-axis listed nodes. Blank squares are those that were pruned during Bayesian model reduction or did not meet the threshold for strong evidence of modulatory connection (> 0.95 posterior probability). The color bar spectrum indicates the normalized strength (magnitude) and the direction (color) of each connection in Hertz. Excitatory (positive) connections being ‘hot-red’ and inhibitory (negative) connections ‘cold-blue.’ For example, the L striatum was excitatory toward the L M1, with greater magnitude than many other connections. M1, Primary Motor Cortex; STN, subthalamic nucleus; L, left; R, right. Each hemisphere model features the contralateral cerebellum.

**Figure 3:**
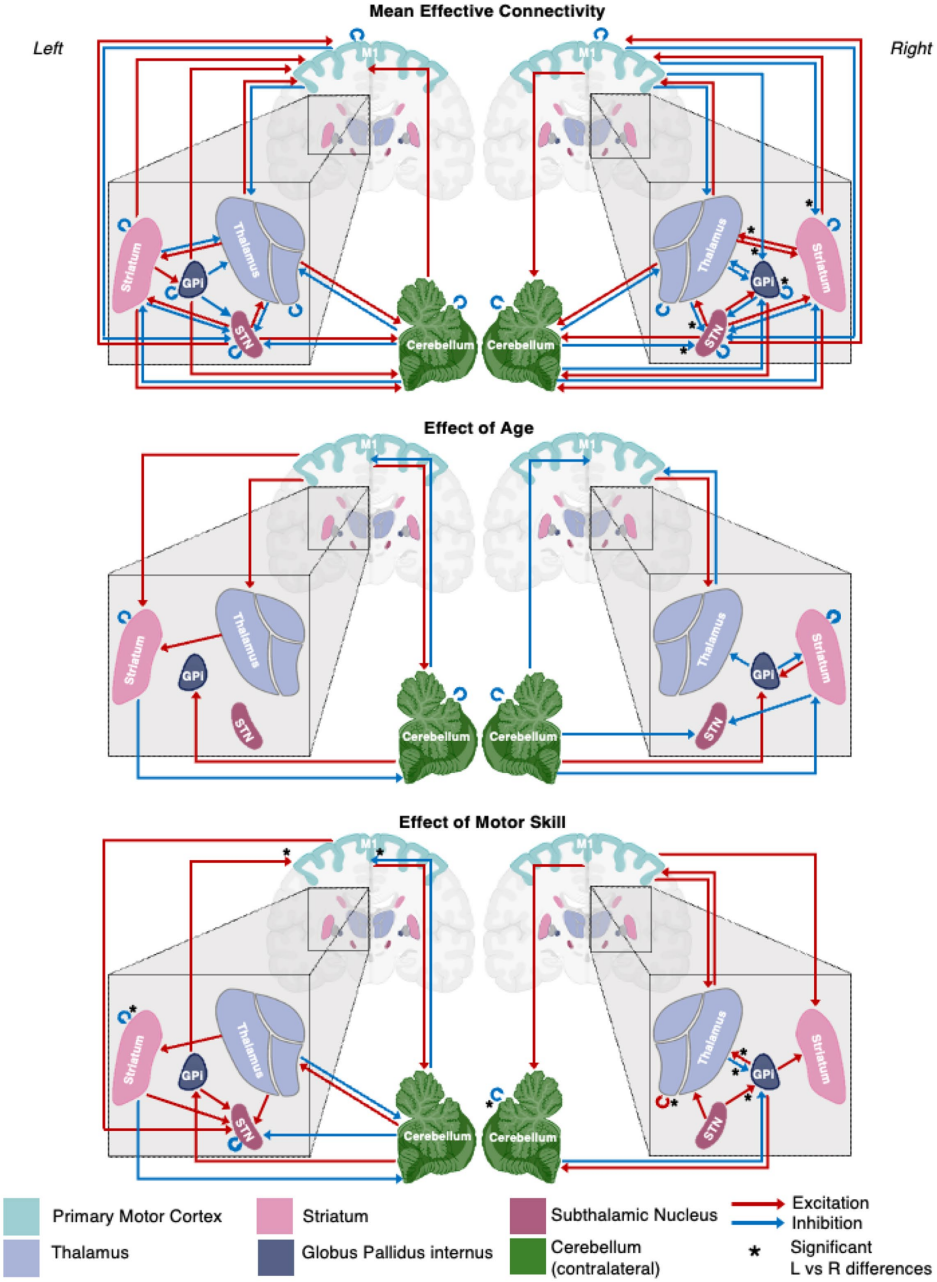
A schematic representation of the motor network mean effective connectivity and the effect of age and motor skill. Arrows represent nodal directed connectivity polarity with red representing negative (inhibitory) and blue positive (excitatory) polarity. Significant inter-hemispheric differences are signaled with a star (*).

The optimized mean EC parameters, passing Bayesian Model Reduction pruning and > 95% probability, are delineated in Figure 2 and summarized in the top panel of Figure 3. Detailed EC parameters and confidence intervals of all parameters can be found in Supplementary Table S2.

#### Primary motor

All surviving modulations toward M1 were excitatory, except for self-modulation. Modulations from M1 were excitatory toward the contralateral cerebellum (right hemisphere model) but inhibitory toward subcortical ROIs (no modulation toward striatum or GPi in the left hemisphere).

#### Striatum

Surviving modulations toward the striatum were primarily excitatory except for self-modulation, from the contralateral cerebellum and, from M1 (right hemisphere only). There was no significant modulation from GPi to striatum in either hemisphere. Modulations from the striatum were primarily positive with the exceptions of toward STN bilaterally and toward the thalamus in the left hemisphere; there was no Striatum➔GPi modulation in the right hemisphere while positive modulation was present in the left.

#### Subthalamic nucleus

Both hemisphere models showed inhibitory modulation toward the STN from all regions with surviving connections. All surviving connections from STN (except self-connection) were excitatory for both hemispheres. There was no STN➔GPi modulation on the left.

#### Globus pallidus internus

Surviving modulations from GPi were mixed, being inhibitory toward STN and thalamus, and excitatory toward the contralateral cerebellum. Modulations from M1, thalamus, and cerebellum were inhibitory (right hemisphere only), while those from STN and striatum (in the left only) were excitatory.

#### Thalamus

Connections from the thalamus survived in the right hemisphere and were mixed in polarity. Thalamus➔GPi did not survive in the left hemisphere, but the polarity of the surviving connections from the thalamus was the same as in the right hemisphere. Striatum➔Thalamus was the only connection that remained in both hemispheres and had opposite polarity (left inhibitory, right excitatory). All other connections to the thalamus were inhibitory.

#### Cerebellum (contralateral)

In both hemisphere models, all surviving connections from the contralateral cerebellum were inhibitory with the exception of M1 which was positive in the left hemisphere and absent in the right. All connections from the contralateral cerebellum to other nodes survived except cCER➔right M1 and cCER➔left GPi. All modulations toward the contralateral cerebellum survived, except for those from the left M1, and were excitatory (except self-modulation).

### Age correlation

**Figure 4:**
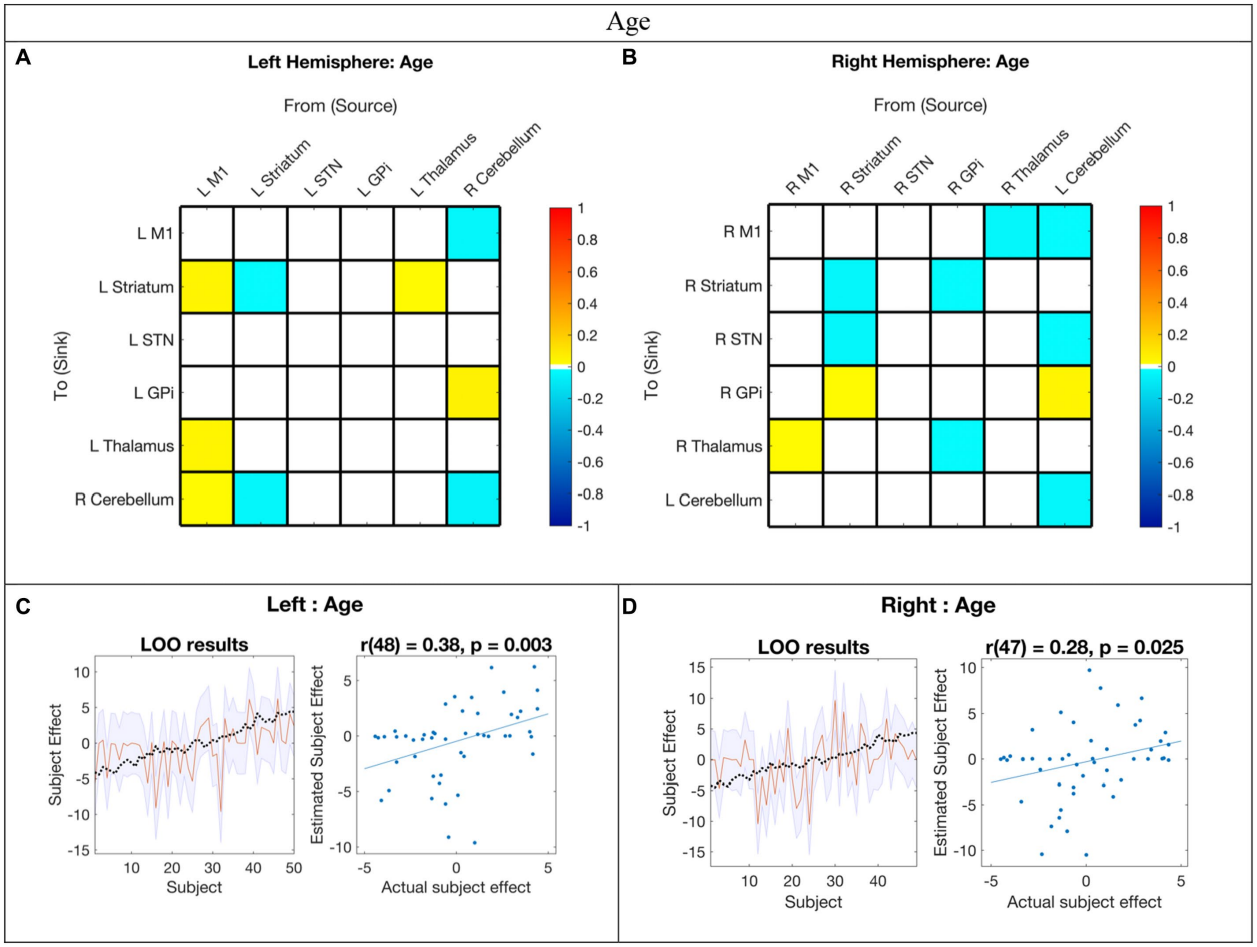
Hemispheric effective connectivity association with age. **(A,B)** Age was covaried with each queried connection. The color bar spectrum indicates the normalized strength and the direction of the relationship between a connection’s value and age. For self-connections, a positive parameter estimate on a self-connection (positive effect of a covariate) indicates a positive relationship between the covariate and the level of self-inhibition and a negative parameter estimate on a self-connection (negative effect of a covariate) indicates a negative relationship between the covariate and the level of self-inhibition. For example, increased age was associated with less striatum self-inhibition bilaterally. For all other connections, positive (hot) parameters indicate that more excitatory/less inhibitory connectivity values are associated with increasing age (positive relationship) and negative (cool) parameter values indicate that more inhibitory/less excitatory connectivity values are associated with increasing age (negative relationship). For example, the right hemisphere M1➔STN connection, greater age associated with more excitation, but for the right hemisphere striatum➔STN connection, greater age was associated with more inhibition. **(C,D)** Leave-one-out cross-validation (LOO-CV) results for each hemisphere. For each LOO-CV test, there are two plots. *Left:* The line graph for each comparison shows the out-of-samples estimate of the mean-centered age (red line) for each subject plus variance (purple shaded area, 90% CI) and the actual group effect (black dotted line). The narrower the purple envelope and the more the black dotted line falls within it and near the red line, the higher the degree of confidence and consistency the brain-age relationship of the tested connections. *Right:* The blue dotted scatter plot compares the actual subject effect to the estimated subject effect of the brain-age relationship for each subject using an out-of-samples Pearson correlation coefficient (r). LOO-CV tests were significant for each hemisphere. M1, Primary Motor Cortex; STN, subthalamic nucleus; L, left; R, right. Each hemisphere model features the contralateral cerebellum.

The EC network parameters evaluated for an association with age with posterior probability >0.95 are shown in Figures 4A,B and summarized in the middle panel of Figure 3. The detailed parameter estimates and confidence intervals of all connections with evidence can be found in Supplementary Table S3.

In the LOO-CV for age, all connections with posterior probability were entered for each hemisphere analysis, respectively. The LOO-CV tests were significant for each hemisphere (left: *r*(48) = 0.38, *p* = 0.003; right: *r*(47) = 0.28, *p* = 0.025, see Figures 4C,D, respectively), indicating that age was not only correlated with these parameters, but predicted by them above chance. However, the results also show that there was still variability not accounted for by age alone in each hemisphere model.

### Motor skill correlation

**Figure 5:**
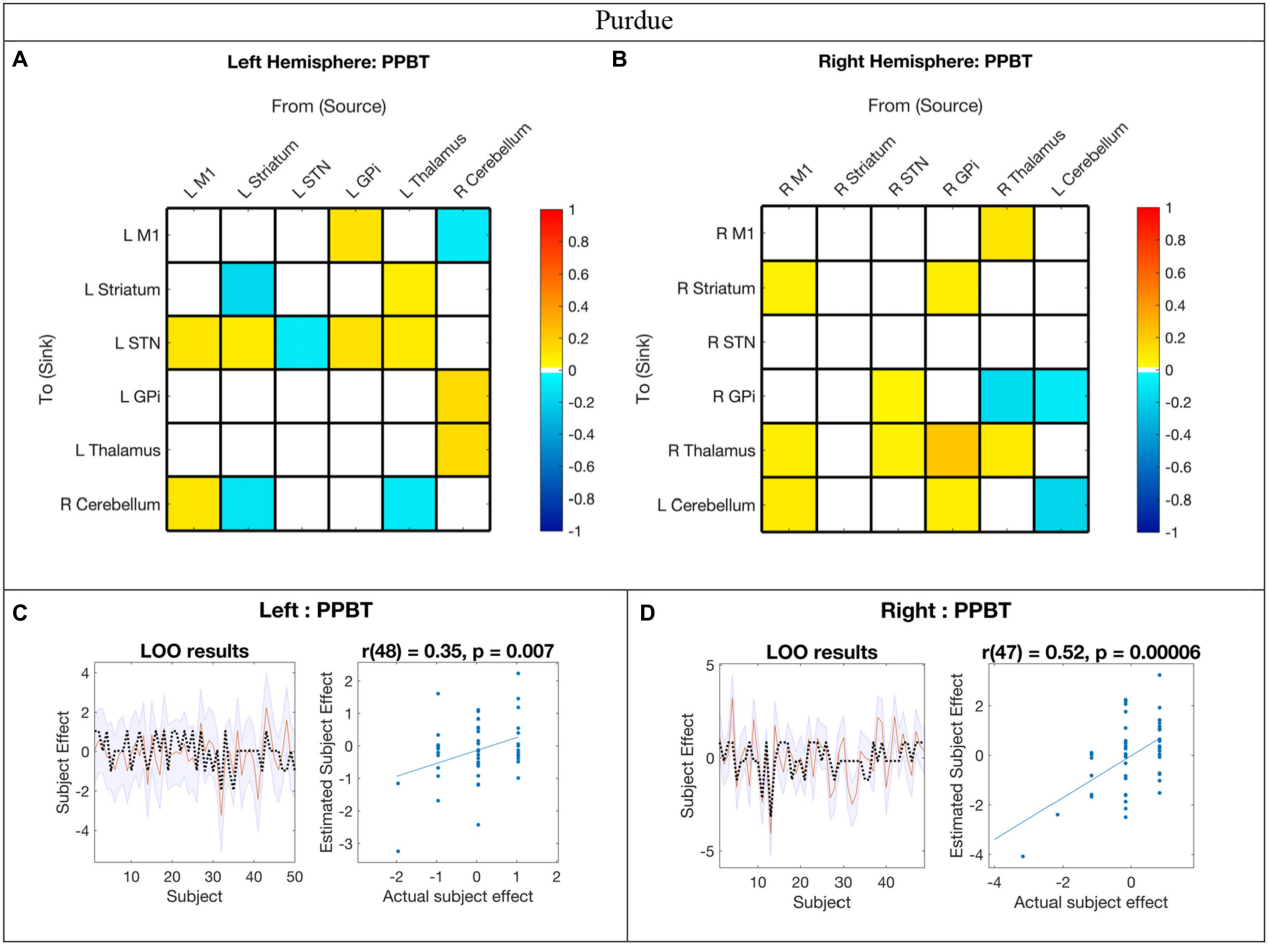
Hemispheric effective connectivity association with Purdue Pegboard Test score. **(A,B)** The Purdue Pegboard Test score (PPBT) for the associated hand (i.e., right hand for left hemisphere, left hand for right hemisphere) was covaried with each queried connection. The color bar spectrum indicates the normalized strength and the direction of the relationship between a connection’s value and PPBT scores. For self-connections, a positive parameter estimate on a self-connection (positive effect of a covariate) indicates a positive relationship between the covariate and the level of self-inhibition and a negative parameter estimate on a self-connection (negative effect of a covariate) indicates a negative relationship between the covariate and the level of self-inhibition. For example, in the right hemisphere, higher PPBT scores were associated with more thalamus self-inhibition, but, for the left hemisphere striatum self-connection, lower PPBT scores were associated with more self-inhibition. For all other connections, positive (hot) parameters indicate that more excitatory/less inhibitory connectivity values are associated with higher PPBT scores (positive relationship), and negative (cool) parameter values indicate that more inhibitory/less excitatory connectivity values are associated with higher PPBT scores (negative relationship). For example, the left hemisphere M1➔STN connection, higher PPBT scores were associated with more excitation, but for the left hemisphere Thalamus➔STN connection, higher PPBT scores were associated with more excitation. **(C,D)** Leave-one-out cross-validation (LOO-CV) results for each hemisphere. For each LOO-CV test, there are two plots. *Left:* The line graph for each comparison shows the out-of-samples estimate of the mean-centered PPBT (red line) for each subject plus variance (purple shaded area, 90% CI) and the actual group effect (black dotted line). The narrower the purple envelope and the more the black dotted line falls within it and near the red line, the higher the degree of confidence and consistency the brain behavior relationship of the given connection. *Right:* The blue dotted scatter plot compares the actual subject effect to the estimated subject effect of the PPBT brain-behavior relationship for each subject using an out-of-samples Pearson correlation coefficient (r). LOO-CV tests were significant for each hemisphere. M1, Primary Motor Cortex; STN, subthalamic nucleus; L, left; R, right. Each hemisphere model features the contralateral cerebellum.

The EC network parameters evaluated for the association with standard deviation PPBT score with probability >0.95 are shown in Figures 5A,B and summarized in the bottom panel of Figure 3. Detailed parameter estimates and confidence intervals of all connections with evidence can be found in Supplementary Table S4.

In the LOO cross-validation for EC-PPBT, all connections with posterior probability >0.95 were entered for each hemisphere analysis, respectively. The LOO-CV tests were significant for each hemisphere [left: *r*(48) = 0.35, *p* = 0.007; right: *r*(47) = 0.52, *p* = 0.00006, see Figures 5C,D, respectively], indicating that PPBT was not only correlated with these parameters, but predicted by them above chance. Similarly, there was still variability in each hemisphere model not explained by motor skill.

#### Hemispheric symmetry

**Figure 6:**
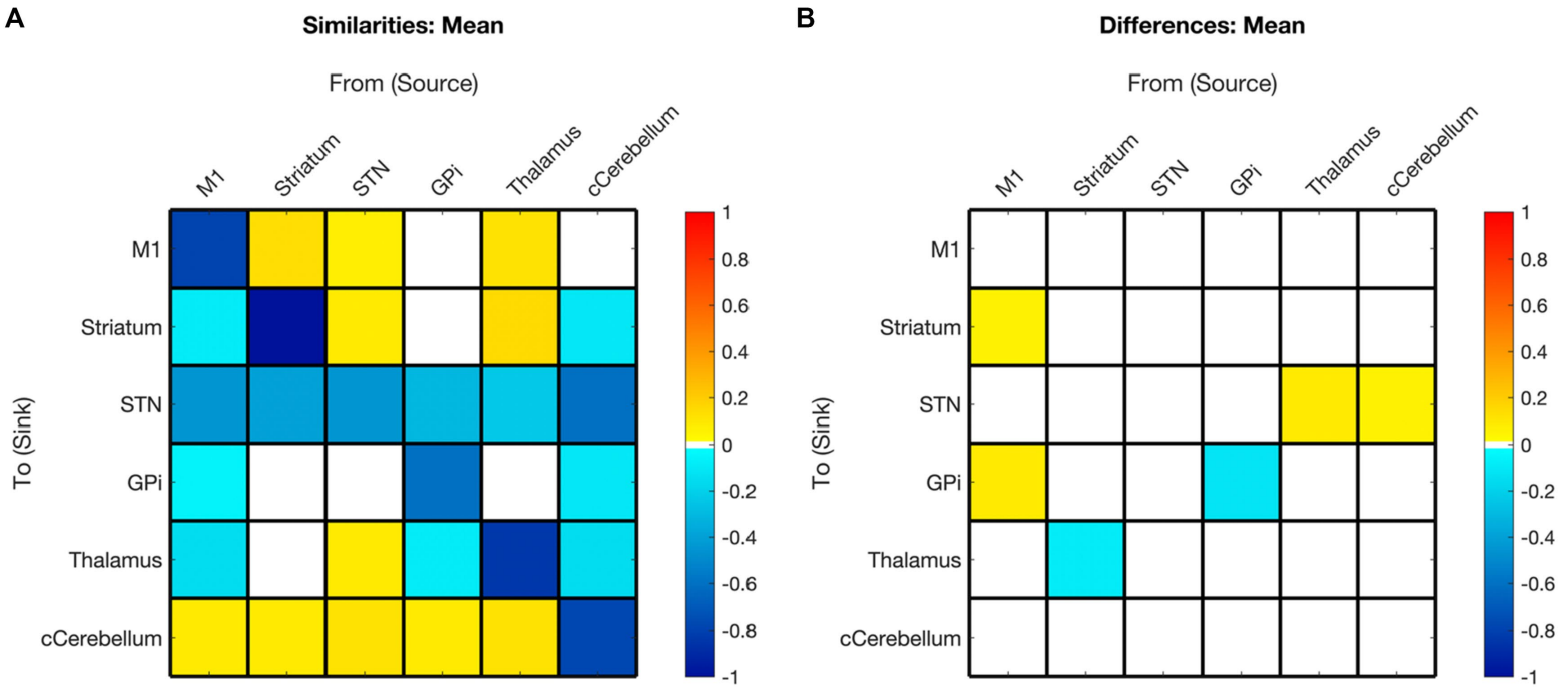
Summary of hemispheric similarities and differences. **(A)** Summary of similarities. Similar parameter estimated are thresholded at >95% posterior probability (free energy). **(B)** Summary of differences. Differences were calculated left minus right hemisphere, therefore, ‘warm’ values (e.g., M1➔Striatum) mean that the value was higher for the left hemisphere than the right and ‘cool’ values (e.g., Gpi➔GPi) mean the value was higher for the right hemisphere than the left. Differences shown are thresholded at 75% posterior probability (free energy). While these differences are reported, the meaning is interpreted with caution, only noting there were far more similarities than differences in these values, implying consistency.

Significant interhemispheric differences were assessed by the hierarchical PEB (PEB of PEBs) shown in Figure 6 and summarized in Figure 3. The left hemisphere had fewer overall connections (29 of 36 total potential connections) than the right hemisphere (32 of 36 total potential connections). Overall, 83% (28/36) of connections had strong evidence (>95% probability) of similarity between hemispheres and 33% (6/36) of connections had positive evidence (>75% probability) of differences. Significant differences included the presence of inhibitory modulation from M1➔GPi and M1➔Striatum in the right (absent in left), stronger inhibition from Thalamus➔STN, cCER➔STN, and GPi➔GPi in the right, and opposite polarity of Striatum➔Thalamus modulation (inhibitory on the left and excitatory on the right).

**Figure 7:**
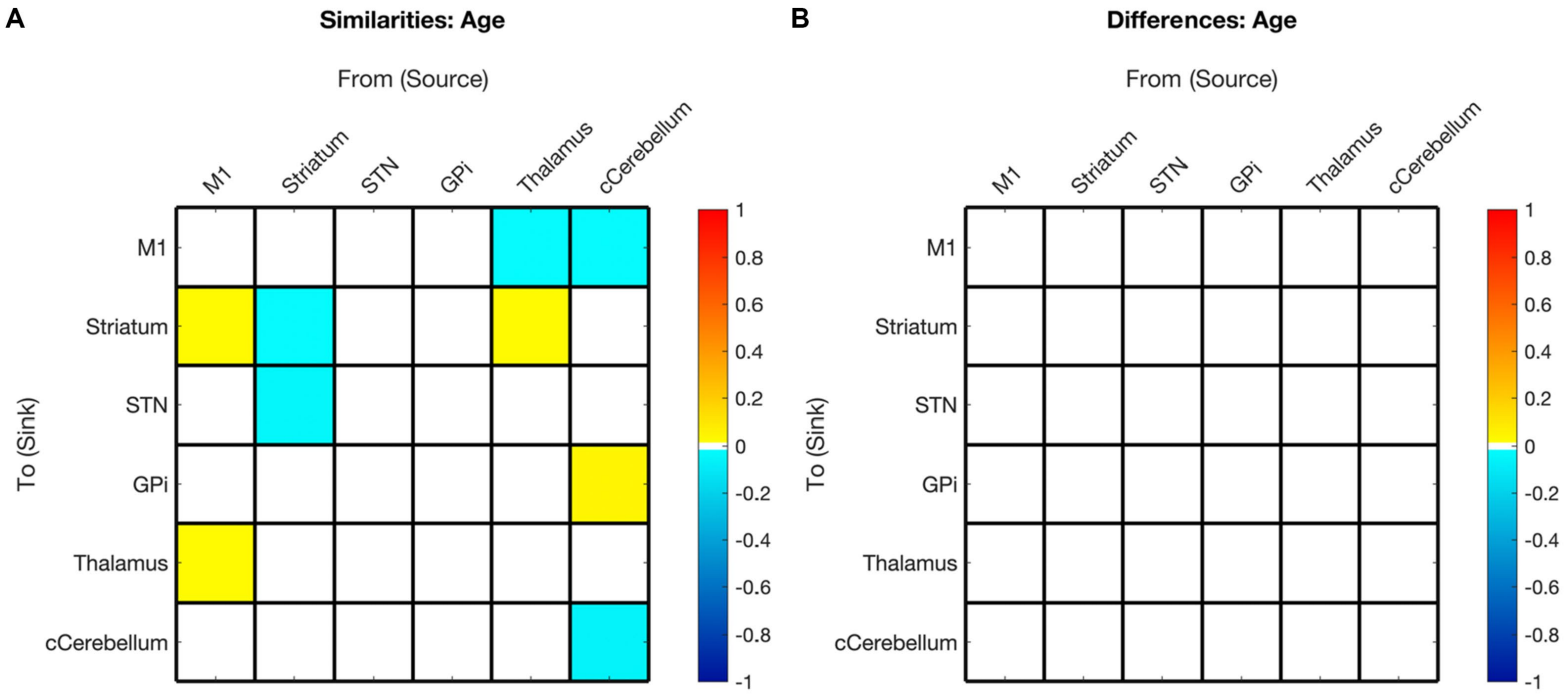
Summary of hemispheric similarities and differences for covaried age. **(A)** Summary of similarities. Similar parameter estimated are thresholded at >95% posterior probability (free energy). **(B)** Summary of differences. Differences were calculated left minus right hemisphere. Differences shown are thresholded at 75% posterior probability (free energy). While these differences are reported, the meaning is interpreted with caution, only noting there were far more similarities than differences in these values, implying consistency. Notably, there are no age differences in this comparison.

**Figure 8:**
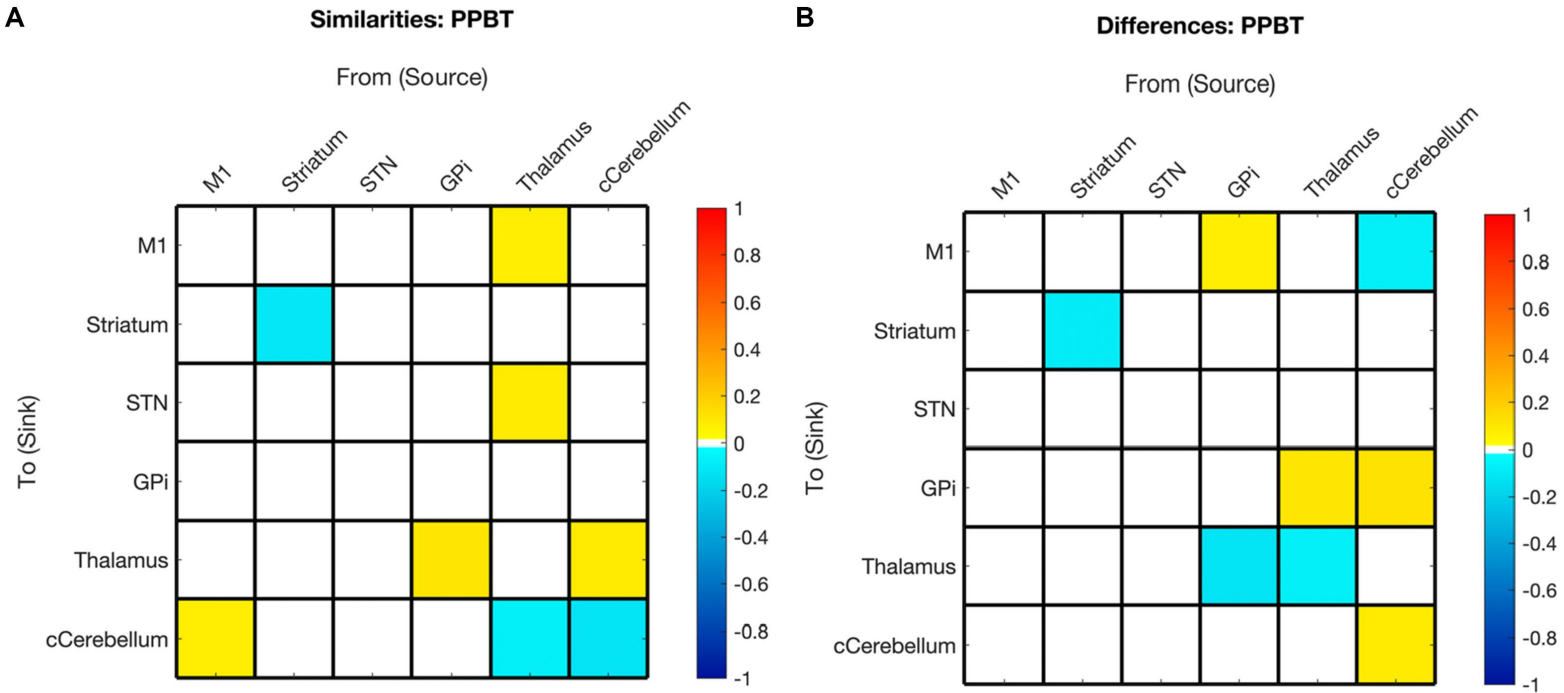
Summary of hemispheric similarities and differences for motor skill as measured by PPBT. **(A)** Summary of similarities. Similar parameter estimated are thresholded at >95% posterior probability (free energy). **(B)** Summary of differences. Differences were calculated left minus right hemisphere, therefore, ‘warm’ values (e.g., GPi➔M1) mean that the value was higher for the left hemisphere than the right and ‘cool’ values (e.g., GPi➔Thalamus) mean the value was higher for the right hemisphere than the left. Differences shown are thresholded at 75% posterior probability (free energy).

The effect of age on the presence and polarity of connections did not show any significant difference when directly comparing hemispheres (Figure 7, Figure 3 middle panel). In contrast, the effect of motor skill showed the largest number of significant differences between hemispheres (Figure 8, Figure 3 bottom panel). These included the presence of inhibitory modulation from Thalamus➔GPi and cCER➔cCER and excitation from STN➔GPi, GPi➔Thalamus, and Thalamus➔Thalamus in the right (absent in left). Connections present in the left that were absent in the right included inhibitory modulation from cCER➔M1 and Striatum➔Striatum and excitatory modulation from GPi➔M1.

A summary of the main and covariate effects for each hemisphere is in Supplementary Table S5.

## Discussion

This report presents the first pediatric rs-fMRI EC study of the MN associated with age and motor skills, which is necessary for meaningful individual comparisons in future EC motor network studies in movement disorders (Sussman et al., 2022). The predictive association between age and EC parameter effect sizes suggests that motor network EC changes with development. Therefore, it is important to consider a developmentally appropriate model and comparisons in children. Additionally, the current results indicate a relationship between motor skill, as measured by the standard deviation of the PPBT score, and EC that is independent of age. Importantly, the association between connectivity patterns and motor skill demonstrated the ability of resting-state models to reflect network dynamics related to motor function. Overall, key patterns of covariate relationships with connections, symmetries, and asymmetries between hemispheres emerged and will be discussed presently.

### Mean effective connectivity and hemispheric asymmetries

While inherent hemispheric asymmetries and specializations are recognized (3,8), fMRI resting state FC is generally understood to be symmetrically distributed across hemispheres (Damoiseaux et al., 2006; Smith et al., 2009). A recent study examining asymmetric resting state FC in adults symmetric connectivity at a 95% level (Raemaekers et al., 2018). As anticipated, there were also great similarities between the left and right hemisphere models in this study, as evidenced by the comparison of the left and right hemisphere PEBs wherein 29 of the 36 possible connections exhibited similarity between hemispheres, and only 6 of the possible 36 connections showed differences.

STN EC connections demonstrated the highest level of consistency across subjects in the mean EC, with the STN being the only node bi-directionally connected to all other nodes. The exception was the STN to GPi connection, which did not survive in the left hemisphere model.

All surviving connections to and from STN were inhibitory and excitatory, respectively. This remarkable consistency and robustness also suggest a high degree of in the role conservation of the STN MN within the motor network, the reproducibility of the EC by DCM, and reflect clinical experiences targeting the STN for DBS in Parkinson’s disease and dystonia (Lumsden et al., 2017). This may underscore the significance of the STN in suppressing unwanted movements (Milardi et al., 2019), particularly in children who are likely awake but instructed to not move.

While previous models anticipated excitation *to* the STN during movement suppression, this study found inhibition, possibly due to the net circuitry inhibition resulted in an overall negative effect of all signals into the STN. In support, an optogenetic fMRI study in mice, using spectral DCM to compare BG circuit function when stimulating D1 versus D2 dopamine pathways, consistently observed either negative or no modulation from the cortex to the STN (Bernal-Casas et al., 2017).

All surviving connections *from* the STN in both hemispheres were excitatory, consistent with prior studies (Haber, 2003). The fact that many of these connections are not direct underscores the capacity of EC to measure dynamic effects across intermediate nodes. The findings of all inhibitory incoming connections and all excitatory outgoing connections for STN are novel.

### The role of GPi and the thalamus

Consistent with previous models of indirect pathway connections, the current study revealed that an increase in GPi activity resulted in a decrease in thalamic activity in both hemispheres (Manza et al., 2015). Furthermore, in line with earlier findings, heightened STN activity led to increased GPi activity (Albin et al., 1989). The GPi disperses the final output of the basal ganglia’s neuromodulatory signal to the thalamus. The GPi’s primary modulators include the STN and the striatum (Albin et al., 1989; Haber, 2003), a relationship reflected in the mean EC model where the GPi received inhibitory modulation from these regions in both hemispheres.

The thalamus’s pivotal role as the final excitatory link from the basal ganglia to the cerebral cortex was consistently observed in both hemispheric models. Additionally, direct excitatory projections to the striatum were identified, aligning with previously described cortical-BG–thalamic loops (Haber and Calzavara, 2009). Notably, the thalamus exhibited several incoming connections in the final model for both hemispheres. Interestingly, each hemisphere displayed five incoming connections to the thalamus with only one of these connections exhibiting opposite polarity (STR➔Thalamus), illustrating predominantly similar but some lateralized motor specialization in the resting motor network EC.

In both hemispheres, the M1 exclusively received excitatory connections and exerted inhibitory modulation toward subcortical regions. Previous models predominantly directed the M1 signal toward the subcortical regions of the STN and striatum, with primary input to M1 originating from the thalamus (Alexander et al., 1986; Albin et al., 1989; Haber, 2003). Many existing models do not include M1 and CER (Rowe et al., 2010; Jahfari et al., 2011; Kahan et al., 2014; Battistella and Simonyan, 2019), resulting in limited understanding of these connections (refer to Kahan et al., 2014; Dirkx et al., 2016; Rothkirch et al., 2018 for models incorporating the cerebellum). Thus, the present study suggests a broader modulatory influence of the M1 within the MN.

All surviving modulations from the contralateral cerebellum were inhibitory in both hemispheres. Neuronal tracing studies in both rodents and non-human primates support connections between the cerebellum and BG, demonstrating evidence of cerebellar projections toward the BG primarily targeting the ‘indirect’ pathway via the striatum and through the thalamus (Hoshi et al., 2005). Additionally, there is evidence of an STN output that targets the dentate nucleus (Bostan et al., 2010).

In the current models, the cerebellum exhibited an inhibitory effect on the thalamus and striatum, indicating a directed (effective) functional connection, which is consistent with studies demonstrating FC between the cerebellar, thalamic, and BG RSNs (Sang et al., 2012). A recent study also revealed EC between the putamen and the contralateral cerebellum during a finger-tapping task; however, this modulation was excitatory (Rothkirch et al., 2018). The inhibitory modulation *observed* in the models in this study may be attributed to differences associated with the resting-state.

Furthermore, there is growing evidence implicating the cerebellar contribution to movement disorders such as dystonia. Rodent models have demonstrated dystonic movement as a result of cerebellar lesions (Neychev et al., 2011), while neurophysiological studies in humans suggest cerebellar involvement in the pathophysiology of dystonia, although conclusive evidence regarding its origin site is lacking (Shakkottai et al., 2017).

### Asymmetries and covariates

In terms of asymmetries, the right hemisphere exhibited a higher overall number of connections, whereas the left hemisphere was slightly more sparsely connected. This disparity may reflect greater specialization, as evidenced by pruning of EC in the left hemisphere, particularly considering that the individuals were right-handed. The striatum was the sole region where outgoing modulations were less abundant in the right hemisphere. The only connection displaying polarity conflict between hemispheres was from STR to the thalamus, which exhibited inhibitory modulation in the left hemisphere and excitatory modulation in the right hemisphere.

All other significant differences between the left and right hemispheres occurred when the connection was present on the right and absent on the left (M1➔STR), or when an inhibitory connection was present in both hemispheres, but the effect size was larger in one hemisphere (M1➔GPi, cCER➔STN, Thal➔STN, GPi➔GPi). However, in comparison to cross-hemisphere differences, the most prominent asymmetries were observed in the effect of covariates, as discussed in the following sections.

### Age-related changes in motor network EC

Our findings suggest that childhood age-related changes in motor EC dynamics exhibit similarities across hemispheres (see Figure 6 and Figure 3 middle panel). Specifically, among the connections that covaried with age, 55% (5/9) in the left hemisphere model were also present in the right hemisphere, and 45% (5/11) of the connections in the right hemisphere were also found in the left hemisphere. Furthermore, all surviving connections that covaried with age in both hemispheres exhibited correlation in the same direction within each hemisphere. Additionally, upon direct comparison of the left versus right hemispheres, none of the differences in connection relationships reached statistical significance, indicating that age may not be strongly associated with differences in EC between hemispheres.

There is a paucity of EC studies exploring hemispheric symmetry, and none that investigate associations with development (Raemaekers et al., 2018). Overall, while it is anticipated that EC undergoes changes with development, it is not unexpected that these changes are largely analogous between hemispheres in right-handed children. Hence, it seems plausible that age-related changes (or lack thereof) in motor EC reflect broader hemispheric symmetries, and that development in these networks, irrespective of motor skills, is predominantly symmetric.

With increasing age, both hemispheres exhibited a weakening of excitatory modulation from cCER ➔ M1 and weakening of inhibitory modulation from M1➔Thalamus and from cCER➔GPi. Modulations that showed strengthening with age included inhibitory self-modulation in the cCER and striatum.

Among the MN nodes, the contralateral cerebellum was the most affected by age-related changes. The cerebellum is suggested to play a central role in adaptive predictive processing owing to its capacity for sequence processing and generation of internal models (Leggio and Molinari, 2015). During infancy, the development of cerebellar white matter pathways is predictive of motor behaviors in toddlers, while perinatal cerebellar malformations are linked to an increased risk for atypical motor development (Wang et al., 2014; Wolff et al., 2017). These findings collectively indicate that alterations in cerebellar connectivity patterns within the MN are pivotal for adaptive motor learning and development.

Connections that exhibited correlation with age and PPBT varied significantly within and across hemispheres. The sole connection in both hemispheres for both covariates was cCER➔GPi. While EC does not necessarily denote a direct structural connection between entities, the correlation between cCER and GPi with age and motor skill align with findings by Milardi et al. (2019), who provided evidence from tractography of a white matter pathway directly linking the dentate nucleus (cerebellum) to various thalamic and basal ganglia structures, including the contralateral GPi. Additionally, Neumann et al. (2015) demonstrated that cerebellum-GPi MEG and local field potential alpha-band coherence at rest were negatively correlated with disease severity of torticollis in adult patients with segmental or cervical dystonia. However, this complexity arises from the observation that all cCER➔GPi connections became less inhibitory with age and PPBT score, except for the right hemisphere PPBT, which became more inhibitory with increasing motor skill. Moreover, the cCER➔GPi connection only persisted in the right hemisphere mean model. It plausible that the absence of the cCER➔GPi connection in the left hemisphere stems from its decreasing inhibition with both age and motor skills. In contrast, the GPi➔cCER connection was excitatory in both hemispheres and was not associated with age or motor skill.

### Relationship between motor skill and EC

In contrast to age, the impact of motor skill exhibited significant variation between hemispheres, with half of the covarying connections displaying correlation in the opposite direction between hemispheres. Specifically, 14% (2/14) of the connections correlated with PPBT in the left hemisphere were also correlated with PPBT in the right, and only one of those connections demonstrated correlation in the same direction. Similarly, 15% (2/13) of PPBT-correlated connections in the right hemisphere were also correlated in the left, with only one connection showing correlation in the same direction.

In addition to the differences in connections correlated in both hemispheres, a notable pattern among PPBT-correlated differences in the left and right hemispheres is that, unlike mean connectivity, PPBT-correlated connections predominantly exhibited a reversed inhibitory/excitatory pattern in each hemisphere. This observation further supports that the notion that the pertinent connections for executing the “same” motor task differ between the dominant and non-dominant hemisphere (i.e., left and right).

This lateralization aligns with findings indicating that putaminal RSNs are more pronouncedly lateralized to the right hemisphere in young children, a tendency that diminishes with age (Agcaoglu et al., 2021). Intriguingly, a recent investigation into handedness and EC in motor systems revealed that right-handed individuals exhibited stronger effective coupling among key left-hemisphere regions compared to the right during unimanual movement, while left-handed individual displayed reduced hemispheric asymmetry overall (Pool et al., 2014).

This inconsistency between hemispheres is likely related to hemispheric dominance and handedness. The left hemisphere is dominant in approximately 90% of right-handed individuals and in 80% of left-handed individuals (Mazoyer et al., 2014). Studies comparing functional connectivity patterns between hemispheres have revealed that hemispheric disparities closely correlate with handedness and language lateralization (Raemaekers et al., 2018). Different motor programs may be employed to complete the PPBT task with the dominant versus nondominant hand. The effective network ‘at rest,’ although similar between hemispheres, may contribute to the representation of different levels of ‘readiness’ or ability to complete the task with different programs.

Furthermore, concerning handedness, hemispheric asymmetries may influence functional specialization. Generally, the left hemisphere specializes in communicative language functions and logical reasoning, while the right hemisphere plays a larger role in spatial reasoning and emotional processing (Sun and Walsh, 2006; Hartwigsen et al., 2021). Regarding skilled action, the left hemisphere is considered to be heavily involved in movement selection and coordination, even for movement of the ipsilateral side of the body (Serrien et al., 2006). In contrast, the right hemisphere, is more engaged in various aspects of motor organization, planning, and execution toward goal-directed behavior (Serrien et al., 2006). Our findings suggest that these distinctions may be reflected, at least in part, by the different EC network connection correlations observed in this study.

One of the notable distinctions between the patterns of PPBT-correlated connections in the left and right hemispheres is that in the left hemisphere, nearly all surviving incoming connections to STN are correlated and become less inhibitory with improving PPBT scores. This observation seems unique to PPBT compared to age in the left hemisphere.

The STN receives direct input from various areas, including the motor cortex, forming the “hyperdirect pathway” of the basal ganglia, which exhibits somatotopic organization toward M1 (Hamani et al., 2004; Emmi et al., 2020), and thalamus (Rico et al., 2010). It also indirectly receives more striatal input via the globus pallidus externus (Emmi et al., 2020). The STN serves as a primary target for deep brain stimulation in dyskinetic movement disorders (Benabid et al., 2009), and children with ischemic or metabolic injuries involving the STN can develop dyskinetic movement disorders, including athetosis and dystonia symptoms of CP. In the context of incoming connections correlated to PPBT in typically developing children, this may be associated with an increased ability to utilize the correct motor programs for the reaching behavior used during PPBT.

It has been suggested that the STN, within the indirect and hyper direct pathways, inhibits or suppresses movements from competing motor programs to achieve a goal (Mink, 1996; Nambu et al., 2002). This notion is further supported the study (Kahan et al., 2014) demonstrating clinical score changes after STN-DBS for Parkinson’s disease were also linked to changes in STN EC.

Most of the other connections in the left hemisphere that are correlated with PPBT involve the contralateral cerebellum, with bidirectional connections between the contralateral cerebellum, both M1, and the thalamus. Several prior studies using other modalities indicate the cerebello-thalamo-cortical pathway’s role in skilled movement in primates (Horne and Butler, 1995). Additionally, fractional anisotropy of the dento-thalamic-cortical tract from the left dentate nucleus to the right DLPFC is associated with earlier rhythm-related finger motor skill (Schulz et al., 2014). Clinically, DBS (Koller et al., 2000) and thalamotomy (Chen et al., 2006) of the region of the thalamus that receives cerebellar input improves the adaptive control of reaching in essential tremor. This suggests that the integrity of cerebellar output toward the thalamus is important for the adaptive control of reaching behavior (Chen et al., 2006), which is used in tasks such as PPBT.

In contrast, since there is not a known direct THAL➔CER anatomical connection that is not mediated by the cortex, the left hemisphere association with PPBT in our study may, at first, seem surprising. However, both thalamic DBS and thalamotomy have been shown to influence EC from THAL➔CER in essential tremor in adults (Gibson et al., 2016; Park et al., 2017). Combined with the current results, this suggests that THAL➔CER modulation, although possibly indirect, is also important for motor ability in neurotypical children. It is plausible that the increased motor skill associated with more inhibitory THAL➔CER modulation is related to different recruitment of the cerebellum for motor tasks and that this relationship is also represented at rest. Of note, in both Park et al. (2017) and Gibson et al. (2016) patients were at rest during fMRI acquisition, thus, the effects seen in our study may be due to the resting-state condition or representative of an indirect connection.

Unlike the left hemisphere, PPBT was not correlated with STN inputs in the right hemisphere—though two outputs were. The pattern of PPBT-correlated connections in the right hemisphere is less clear. While right PPBT-EC certainly is not limited to it, it is interesting that, unlike the left hemisphere, a portion of correlated connections fall along direct connections in the classic basal ganglia/movement pathways and these were unique to the right hemisphere (specifically: M1➔striatum, STN➔GPi, GPi➔Thalamus, and Thalamus➔M1).

The differences in connectivity between GPi and M1 across hemispheres were another finding of potential importance in motor control. The GPi projects out from the BG to the thalamus and ultimately to the motor cortex, and its outputs have been associated with movement disorders (Neychev et al., 2011; Milardi et al., 2019). In the dominant left hemisphere, the GPi exerted excitatory modulation toward M1 which increased with higher motor dexterity. Meanwhile, in the right hemisphere, an inhibitory M1➔GPi modulation was observed.

Another interesting hemispheric difference was the correlation between the increased excitatory signal for Thalamus➔STR with PPBT score in the left hemisphere. This is consistent with the finding that thalamostriatal projections are associated with skilled initiation of movement sequences in mice (Díaz-Hernández et al., 2018).

Overall, the significant brain-behavior relationship elucidated in this study with a relatively small variability in normal motor behavior, in a narrow age range, and relatively small sample size, is indicative of this measure’s potential in comparing subjects with gross pathology to normal. Furthermore, PPBT scores were, in general, not correlated with the same parameters for each hemisphere. This is something that should be taken into consideration when describing the relationship between clinical scores and potential neuroimaging biomarkers. Furthermore, not until rs-fMRI EC and PPBT in children with CP and DBS candidacy with outcomes, will we determine if such EC-motor skill relationship is helpful.

### Model comparisons

This EC MN model exhibits several fundamental differences compared to prior work, and as such, the disparities are to be anticipated.

1. The EC model includes *more subcortical ROIs* than some MN models (Kahan et al., 2014; Diekhoff-Krebs et al., 2017; Battistella and Simonyan, 2019; Zhang et al., 2020), as a broader perspective of the MN dynamics was desired, and rs-fMRI did not impose relevant spatial limits.
2. Unlike most MN studies conducted under active or (Jahfari et al., 2011; Rothkirch et al., 2018) passive tasks [e.g., stimulation, tremor onset conditions (Kahan et al., 2014; Dirkx et al., 2016; Bernal-Casas et al., 2017)], the subjects herein were instructed to *rest without motion* for 5 min, theoretically requiring either continuous impulse inhibition and/or a state of executively sanctioned relaxation.
3. In 5- to 13-year-old children told to be perfectly still, it is possible that this may result in “*active” stillness*, wherein instead of relaxing, intermittent stiffening or any number of difficult-to-visualize actions to maintain stillness is possible. This could have consequences for the network dynamics.
4. The direct, indirect (Albin et al., 1989), and hyperdirect (Nambu et al., 2002) pathways were not explicitly modeled (e.g., Jahfari et al., 2011; Kahan et al., 2014; Dirkx et al., 2016; Bernal-Casas et al., 2017). Instead, this EC model makes *no assumptions on which nodes are effectively connected, nor how*. This method is based on the relatively well-established premise that when two nodes are not immediately connected, they could still influence each other. This *more data-driven* facet of the EC model was intentional because the conditions of the data acquisition had no prior established precedent. Thus, novel network dynamics of this condition may be discovered.
5. Prior models largely did not include a query for *bidirectional signal* (e.g., both forward and backward directed connections) (Kahan et al., 2014; Dirkx et al., 2016), whereas the study EC model did make this allowance. This is key, because if signal from A to B is the only direction queried, then B to A will remain unknown. Including feedback or feedforward connections may change the results of the system dynamics. It may be argued that only previously established connections should be modeled. However, within the non-independent parallel loop system of the MN, all such EC are biologically plausible. Further, as a loop, all members’ activity should or could have direct or downstream results on all other members.
6. Developmentally related EC MN differences compared to adults are unknown, thus any portion of the differences herein could be attributed to age.
7. Many prior models only investigated a single hemisphere or did not explicitly consider hemispheric differences (Kahan et al., 2014; Dirkx et al., 2016; Rothkirch et al., 2018), thus differences in the direction of network signal are less well established.

### Future directions

It is of interest to compare the dynamic motor network features across the lifespan and disease severity continuum. As such, the current normally developing children’s results herein are intended to be utilized in a comparison with age-matched children with motor deficits. In the future, the normative EC will be extended through the remaining age range for neurotypical children. An equivalent study in adults is also needed to understand the trajectory of network maturation. Further validation of the current study’s findings in a larger sample would increase understanding of reproducibility and reliability.

Regarding regions of interest inclusion, increasing regions of cortex, and reducing regions of cerebellum to motor nuclei are reasonable considerations. One study in animals showed contralateral cortical to striate connections, not evaluated in our study, with implications in compensatory mechanisms with lesion, it is also a consideration to add such regions to the model moving forward (Borra et al., 2022). Additionally, we opted to include the entire cerebellum, rather than specific cerebellar motor regions, as broader areas are involved in motor learning (Baladron et al., 2023). However, such narrowing is a consideration for future studies. Lastly, it is also a consideration to include the premotor cortex, which has extensive connections not only to the STN but also with the striatum (Bufacchi et al., 2023).

## Limitations

Since this study includes only right-handed children, generalizations to left-handed or ambidextrous children should be made cautiously, and require further specialized study to make inferences with atypically handed children with CP. The data in this dataset also contain relatively short rs-fMRI sessions, which carries a higher concern for noise. At the time the data were acquired, the C-MIND database was the only publicly available dataset with healthy, neurotypical, young children that also included hand dominance and motor scores. Future studies may expand to a larger age spread to build developmental trajectory, include left-handed participants to identify any differences, and compare clinical groups, such as children with movement disorders who can be completed under conscious sedation with surgically reliable signal acquisition in children (Boerwinkle et al., 2018a,b, 2019). Additionally, the relationships between PPBT score, and parameter estimates may be different in children with movement disorders compared to typically developing children. Future studies should investigate this possibility.

## Conclusion

In this study, we used spectral DCM with a PEB approach to identify a pattern of typical directed effective connectivity in healthy 5- to 13-year-olds in the motor network consisting of cortical, subcortical, and cerebellar locations as well as associations with motor skills. Bayesian Model Reduction and Bayesian Model Averaging led to similar models of intrinsic connectivity for both hemispheres, even when each hemisphere was separately specified, estimated, and reduced—providing evidence of reliability for this technique. We demonstrated that using resting state fMRI can capture EC MN differences that are related to motor skills. Overall, more connections were present in the right, while motricity exerted a stronger effect over connections on the left hemisphere, suggesting the emergence of specialized pathways within the dominant hemisphere with increasing age and motor skill. While prior task-based fMRI methods allow for more direct testing of established motor action pathways, rs-fMRI is often more plausible to collect in children, particularly those with complex movement disorders. Establishing models of normative directed connectivity in a resting-state has the potential to improve precision for treatments in complex movement disorders.

## Data availability statement

Data and/or research tools used in the preparation of this manuscript were obtained from the National Institute of Mental Health (NIMH) Data Archive (NDA). NDA is a collaborative informatics system created by the National Institutes of Health to provide a national resource to support and accelerate research in mental health. Dataset identifier(s): 10.15154/1524281. This manuscript reflects the views of the authors and may not reflect the opinions or views of the NIH or of the Submitters submitting original data to NDA.

## Ethics statement

The studies involving humans were approved by Phoenix Children’s Hospital Institutional Review Board. The studies were conducted in accordance with the local legislation and institutional requirements. The human samples used in this study were acquired from publically available research database. Written informed consent for participation was not required from the participants or the participants’ legal guardians/next of kin in accordance with the national legislation and institutional requirements.

## Author contributions

VB: Conceptualization, Formal analysis, Funding acquisition, Investigation, Methodology, Project administration, Resources, Supervision, Writing – original draft, Writing – review & editing. BS: Conceptualization, Data curation, Formal analysis, Investigation, Methodology, Visualization, Writing – original draft, Writing – review & editing. LL: Conceptualization, Formal analysis, Visualization, Writing – original draft, Writing – review & editing. SW: Writing – review & editing, Conceptualization. WR: Visualization, Writing – review & editing. MK: Writing – review & editing. MA: Writing – review & editing. JF: Conceptualization, Software, Writing – review & editing.
