# Supplementary material for: Motor network dynamic resting state fMRI connectivity of neurotypical children in regions affected by cerebral palsy
Source: Front Hum Neurosci. 2024 May 21;18:1339324. doi: 10.3389/fnhum.2024.1339324 (PMC11148452; doi:10.3389/fnhum.2024.1339324)
Supplement: Supplementary file 1 [file Table_1.pdf]

Supplementary Table S1. Individual Parameter Estimates and converted Purdue Pegboard Test Scores

	Left Hemisphere																	
	sd-PPBT	Source: M1						Source: Striatum (STR)						Source: Subthalamic Nucleus (STN)				
Subject	Right Hand	Left M1-M1	Left M1-STR	Left M1-STN	Left M1-Gpi	Left M1-Th	Left M1-cCER	Left STR-M1	Left STR-STR	Left STR-STN	Left STR-Gpi	Left STR-Th	Left STR-cCER	Left STN-M1	Left STN-STR	Left STN-STN	Left STN-Gpi	Left STN-Th
Column 1	Column 2	Column 3	Column 4	Column 5	Column 6	Column 7	Column 8	Column 9	Column 10	Column 11	Column 12	Column 13	Column 14	Column 15	Column 16	Column 17	Column 18	Column 19
1	0	0.497277608	-0.178684131	-0.349359091	-0.402618102	-0.323059734	0.035004229	0.057094475	0.507760903	0.907564225	-0.45602019	0.232930116	0.110416173	0.09187736	-0.01234162	-0.183825204	-0.215564614	0.016271726
2	0	0.50639694	-0.094016815	-0.213649525	0.547259761	-0.63092877	0.125359045	0.265331072	0.723142265	-0.332206929	1.089896463	-0.283199335	0.31141332	0.091618362	0.102589868	-0.094719004	0.145217886	-0.122571982
3	-1	0.432973943	-0.090408293	-0.559884059	-0.119916997	-0.013964626	-0.598090526	0.254656852	0.805939688	-0.204034485	0.133332615	0.213810519	-0.128394383	-0.137379045	0.036037998	-0.0618141	0.059690375	0.048191601
4	0	0.483112053	-0.077562067	-0.159899003	-0.020644497	0.147460629	0.164340285	0.091114728	0.49317024	-0.673622954	-0.291146693	0.136981946	0.19663281	0.025629147	0.135288292	-0.19641334	0.106204765	0.160868424
5	-2	0.465822477	-0.009957845	-1.017498991	-1.197972283	0.242362349	0.040458906	0.239477804	0.879201984	-0.735636163	-0.412857985	-0.095107802	0.282991565	0.036428164	0.15232669	-0.055780782	0.074810005	0.115981072
6	-1	0.438683655	0.049236426	0.000294508	-0.124297589	-0.238204351	0.076115542	-0.29385132	0.841498876	-0.373146224	-0.117574244	-0.302503283	0.109439628	-0.030003316	0.070363597	0.119447539	-0.003492511	-0.041622295
7	-1	0.508956686	-0.103573394	-0.827920596	-0.011097379	-0.313323092	0.113820412	0.077719048	0.991477892	-0.344531951	-0.13664237	-0.321708495	0.313282383	0.078279004	0.07690139	-0.044139824	0.168976934	0.120460765
8	-1	0.488665649	-0.030221582	-0.687934402	1.172389323	-0.149234287	0.012940574	0.330915708	0.623340413	-0.731277351	0.729760313	-0.035659168	0.198798071	0.094108745	0.169823812	-0.092756246	-0.113647731	0.127311031
9	0	0.497965553	0.050845738	-0.12298667	0.027401503	-0.63201472	0.275981501	0.198786123	0.807534188	-0.343633417	-0.075955558	-0.123204699	0.316599017	0.041465371	0.117508274	0.048962372	0.09720741	0.058066451
10	-2	0.548649455	-0.092107642	-0.34769887	-0.234101142	0.3772263	-0.242127017	-0.007425928	0.848425594	-0.403543004	-0.348511459	-0.491247752	-0.022894849	0.100726263	0.155948726	-0.149258428	-0.08985626	0.103007939
11	-1	0.409352646	0.079663507	-0.659615525	0.237983151	-0.259245793	0.088108026	0.45602254	0.668346197	-0.568383318	0.053776959	0.08467111	0.385701539	0.107101987	0.187018926	-0.281362074	-0.014613995	0.2118843
12	0	0.51098517	-0.183088785	-0.590139567	0.279586466	-0.339797524	0.095646965	0.104025665	0.76522016	-0.672013119	0.189310361	0.411577385	0.288707646	-0.004626347	0.20771024	-0.161248031	-0.02888341	0.05315752
13	-1	0.462571808	-0.161355027	-0.930170794	0.720648282	-0.393151052	0.301159444	0.020087782	0.627122903	-0.264530968	0.535843005	-0.27811668	-0.189446752	0.291722814	0.109963435	-0.062477821	0.393524817	-0.046977876
14	-2	0.463924492	-0.063013106	-0.28861109	0.279140193	-0.781808521	0.06989129	0.123098807	0.763574366	-0.395293059	-0.226982407	-0.070956302	0.462390369	0.028690318	0.09378794	0.087185746	0.144179809	0.031301812
15	-1	0.465020678	0.03302592	-0.640333222	0.232371539	0.120283998	0.10170306	0.187884379	0.700108151	-0.478877631	0.487528049	0.119205392	0.246266169	0.129014164	0.184854833	-0.120558624	0.247653366	0.177874941
16	0	0.506244888	-0.242081863	0.027815867	-0.123491952	-0.007361682	0.102328711	0.103431336	0.395493278	-0.642541024	0.894383154	-0.31588495	-0.055406244	-0.061308091	0.116949201	-0.09419365	-0.200322647	0.004754308
17	-1	0.470283017	0.033084158	-0.993232753	-0.126337109	-0.538948725	-0.251518143	0.280402134	0.856526884	-0.557477732	-0.185181291	-0.13544902	0.031280371	0.136383983	0.147961367	-0.048212765	-0.006351317	0.151594473
18	0	0.48329911	-0.078536477	-0.57588325	-0.152248937	-0.116098211	0.179217197	0.106520304	0.954666063	0.094229384	0.122176209	0.032891083	0.154994069	0.1564712	0.253900489	-0.297484133	0.179885751	0.213578866
19	-2	0.48008318	-0.177750104	0.100735576	-0.619804556	0.059947315	-0.069181151	0.055350848	1.091417516	-0.401641153	0.046932455	-0.200002798	0.019973454	-0.025797341	-0.035637697	-0.096756576	-0.201252847	0.015110951
20	0	0.441908026	0.02765283	0.07452002	-0.002574865	0.002151232	0.208966102	0.127988543	0.37281525	-0.496505422	-0.260493226	-0.309378817	0.034303327	-0.008378093	0.216846471	-0.069596247	-0.103588606	-0.01354116
21	0	0.473680492	-0.107274064	-0.080323795	-0.286310902	-0.366342153	0.028027795	0.077290236	1.020638964	-0.173000599	-0.000994019	-0.202018085	0.166273871	-0.029419125	0.064564816	-0.245328546	-0.008000629	0.049236564
22	-1	0.43581906	-0.129071224	-0.935929564	-0.374897831	-0.923779043	0.465156597	0.308930247	0.81053388	-0.738412294	0.187206675	0.169278734	0.359676338	-0.014355248	0.275476397	-0.246690748	0.097997353	-0.001272945
23	0	0.451904938	-0.034567868	-0.942666173	0.597675022	-0.860466574	0.000492187	0.34070396	0.840231888	-0.812614447	0.358741604	-0.360796731	0.033123627	0.124754349	0.1257815	-0.136278334	0.036102135	0.060550083
24	-1	0.465273949	-0.019117636	0.031422135	-0.07444472	-0.104402742	-0.343009377	0.143004211	0.06721602	-1.189939546	0.894883419	0.121445976	-0.30978693	-0.028682555	-0.140922093	-0.076159677	-0.171733337	-0.096750083
25	0	0.48999354	0.042770328	-0.40199213	-0.324093681	-0.488085015	0.136852642	0.175708119	-0.014155707	-0.653626539	-0.954232492	-0.250452334	-0.0726293	0.129785829	0.012752382	0.023410673	-0.00729778	0.108306313
26	-2	0.499466959	0.09320684	0.054788046	0.620079129	-0.059793065	-0.160005728	0.215413199	0.776012396	-0.576948541	0.509809165	-0.375890655	-0.197894848	0.058058614	0.183677293	-0.09123876	-0.006456552	0.077543413
27	0	0.446706825	0.113739317	0.118717857	-0.266477106	0.093022584	-0.496896314	0.180654936	0.206170952	-0.436768985	0.102469062	0.536648592	-0.707908809	-0.112594539	-0.026972846	-0.194641672	0.002965513	0.043933437
28	-2	0.479284693	0.042128845	-0.952601566	0.188430835	0.699293969	0.188362482	0.158013132	0.799133979	-0.757085097	0.178680696	0.466116491	0.2932116	0.149799772	0.198534356	-0.037262156	0.228176411	0.337644155
29	-1	0.459556514	-0.13157631	-0.48207088	0.512758829	-0.637022049	0.188205443	0.130242174	0.952607921	-0.446189674	0.033865684	-0.405841248	0.11524481	0.104701702	0.104153243	-0.05657974	0.209602997	0.033534122
30	-2	0.545984333	0.06450257	-0.318913929	0.188316673	-0.102983592	-0.109983491	0.005674826	0.275185024	-1.366440734	0.893643609	-0.11090451	0.109791955	0.01695897	0.2677623	0.077243172	0.300270527	0.010224821
31	-1	0.498329081	-0.110873843	-0.292907793	-0.332768935	-0.209829021	-0.104192587	-0.141137761	0.831682953	-0.183981577	-0.284241918	0.20127567	0.032905331	0.107439259	0.14976637	-0.202311988	-0.007213901	0.226678401
32	-3	0.39840044	-0.092425884	-0.591667006	-0.305202562	-0.761385277	-0.150670328	0.093644988	0.932349145	-0.51784254	-0.207962994	-0.055832887	0.319839657	0.032136523	-0.022724544	0.0576931	0.109248515	0.05028685
33	0	0.490707063	-0.133930076	-0.776530415	0.459161698	-0.542004965	0.281609581	0.033109456	1.057607521	-0.541721923	0.489074076	-0.214470298	0.170436318	0.083608395	0.065547118	-0.030023906	0.238971899	0.063881572
34	-2	0.454607687	-0.014978182	-0.929233926	0.409716291	-0.32881371	0.164398455	-0.123595964	0.754968332	-0.290100204	0.499115644	-0.282085822	0.378975533	0.145931875	0.075724211	-0.189613833	0.046750526	0.141873782
35	-3	0.508905686	-0.083535547	-0.712469644	-0.778376194	-0.457249571	-0.228347524	-0.020982708	0.875057887	-0.437238739	-0.227305222	-0.223772095	-0.047819944	0.014778611	0.021777638	0.074699429	0.061003786	0.003336108
36	-1	0.485035732	0.111931939	-1.094261343	0.225298851	0.226212963	0.313155356	0.328871958	0.616556696	-0.415142538	0.265820859	0.125702516	0.255011682	0.147690795	0.117077468	-0.283570589	0.121740312	0.15142072
37	-1	0.475453573	-0.015157123	-0.625475533	0.250842762	-0.363837079	0.172132303	0.262942051	0.631352487	-0.56342967	0.443496587	-0.789365187	0.24533649	0.109050292	0.054330443	0.062109161	-0.001015866	0.066379198
38	-1	0.472087641	-0.05407006	-0.994080394	-0.543398493	0.193883194	0.066667711	-0.051032947	0.749044871	-0.604576417	-0.420832069	0.159525846	0.089126605	0.10193275	0.009630451	-0.056164289	0.142932671	0.051250067
39	-1	0.466101764	0.107767871	0.081073189	-0.007278687	0.196270622	0.316698308	-0.060373379	0.893577525	-0.639350193	-0.355919872	-0.240298157	0.649108978	0.129589417	0.015226744	-0.257761018	0.215624163	0.077730448
40	-1	0.509050553	0.034283531	-0.403184338	-0.367643769	0.055465766	0.379590798	0.119696699	0.667121204	-0.263695586	-0.045734572	0.074004124	0.630935676	0.050598523	0.108853145	-0.014170874	0.040971898	0.14764336
41	-1	0.457999463	0.213353252	-1.220903212	-0.387695414	0.925759718	-0.280134527	0.487604103	0.556485191	-1.219629364	-0.177882493	0.746944697	-0.454560055	0.173415108	0.217060127	0.012359082	0.080568768	0.255047291
42	-2	0.536358128	-0.066031137	-0.475853954	-0.301580828	-0.060827204	0.112705863	0.057852466	0.940664123	-0.145510802	-0.017699398	-0.101891875	0.072898165	0.030390203	0.050549499	-0.18816483	0.195342539	-0.044971604
43	0	0.518301049	-0.005007057	-0.445485432	-0.161682829	-0.064292354	-0.049909826	0.112247447	0.276761201	0.059691995	0.124693239	-0.671834123	-0.845602756	0.044912989	0.32652942	-0.193016491	0.047998043	-0.079540596
44	-1	0.441984835	0.061740917	-0.796995912	0.089676281	-0.674244789	-0.170208008	0.219351057	0.189523529	-0.307547303	-0.730551887	-0.671769859	-0.643620939	0.128494071	0.179140104	-0.077330541	-0.017940498	0.080968742
45	-1	0.468988679	-0.017079487	-0.542514331	0.521165195	-0.443146455	0.182344653	0.231371637	0.545275598	-0.588879536	0.223936371	0.125134413	0.269115821	0.028295766	0.054716622	-0.075647014	0.010805321	0.169037721
46	-1	0.478971748	-0.028798856	0.026773576	0.601325919	-0.081485753	0.430931736	0.084788938	0.835005681	-0.734975445	0.034445532	-0.064632269	0.417359359	-0.00373864	0.236795435	-0.145856752	0.143417412	-0.099081946
47	-2	0.441636738	0.066720778	-0.471979369	0.216346217	-0.240337411	0.405301128	0.378380591	0.329232336	-0.73227076	0.420898528	-0.255580756	0.291608901	0.135504897	0.194544601	-0.151364473	0.03373738	0.302476578
48	-2	0.531843161	0.021200666	0.182052512	-0.539528797	-0.130951829	0.029386798	0.214895021	0.744797454	0.312123595	-0.581719693	-0.002753768	0.1172082	0.047558602	0.116825775	-0.19747549	-0.397194442	-0.097124925
49	-1	0.499438194	0.115578063	-0.664945816	0.707222584	0.29895197	-0.125848915	0.318983265	0.739253934	-0.801789303	0.494205867	0.041518557	-0.302695961	0.106449609	0.158878193	-0.004085436	-0.182253582	0.149174523
50	-2	0.501941787	-0.009028979	-0.923039432	0.534265113	-0.183550579	0.244557489	0.169576906	0.87849898	-0.581081776	0.440275809	-0.154415888	0.28021106	0.097382965	0.09943333	-0.07686401	-0.021224024	0.120243087

	Left Hemisphere																		
	Source: Subthalamic Nucleus (STN)	Source: Globus Pallidus Internus (Gpi)						Source: Thalamus (Th)						Source: Contralateral Cerebellum (cCER)					
Subject	Left STN-cCER	Left Gpi-M1	Left Gpi-STR	Left Gpi-STN	Left Gpi-Gpi	Left Gpi-Th	Left Gpi-cCER	Left Th-M1	Left Th-STR	Left Th-STN	Left Th-Gpi	Left Th-Th	Left Th-cCER	Left cCER-M1	Left cCER-STR	Left cCER-STN	Left cCER-Gpi	Left cCER-Th	Left cCER-cCER
Column 1	Column 20	Column 21	Column 22	Column 23	Column 24	Column 25	Column 26	Column 27	Column 28	Column 29	Column 30	Column 31	Column 32	Column 33	Column 34	Column 35	Column 36	Column 37	Column 38
1	0.10547086	-0.060312173	0.623314966	-0.837685547	0.051676166	-0.028066375	0.051324981	0.177028049	0.188365111	0.241456445	-0.148777703	0.587858856	0.203591765	0.222545942	-0.160291954	-0.08575744	-0.247496186	-0.007323794	0.812944777
2	0.008193094	0.323346464	-0.14360191	-0.249736982	-0.184145147	-0.602887361	-0.020016585	0.235239081	0.237020592	-0.043370766	0.204103658	0.32937049	0.219830281	0.424758002	-0.193159292	-0.08927337	0.603811449	-0.732791728	0.389826819
3	0.247330812	0.349735359	0.310091416	-0.417921331	0.347259059	-0.185959114	-0.182551606	0.193180063	0.075058524	0.227422901	0.090381264	0.577254584	-0.011022588	0.07338117	0.244064838	-0.268002159	0.379682773	0.007572969	0.230551192
4	0.059880811	0.078510714	0.266846362	0.530405958	0.091456448	-0.009435158	0.307074962	0.218327364	0.055123274	-0.708842138	0.210283291	0.553680119	0.173829685	0.173182076	0.209463496	-0.565597987	-0.318902348	0.13182457	0.743221141
5	0.155811536	0.133008857	0.244909394	0.056172515	0.149222804	-0.023631022	0.156501887	0.101628497	0.001946042	-0.641097584	-0.529697781	0.593631044	0.029233892	0.250218316	0.020908008	-1.15944639	-0.478768695	-0.037809202	0.487657581
6	0.031351155	0.044678509	-0.139173486	-0.038748024	0.240790554	-0.282874543	0.023324603	0.218206888	0.093589304	-0.452941512	0.152173022	0.381382237	0.131467945	-0.095141114	0.051508689	-0.314074446	-0.579159843	-0.436667693	0.842279851
7	0.12084745	0.208101199	0.019618935	-0.501274587	0.075234148	-0.396727385	0.304698748	0.135119987	0.041930219	-0.242682523	0.056959745	0.676331788	0.020001174	0.159718701	-0.201715227	-1.108508397	-0.356968884	-0.080293564	0.453853202
8	0.060479783	0.098855431	-0.33496594	-0.622305639	-0.614308236	-0.187913707	0.043041864	0.149757251	0.167473324	-0.357002369	0.175222825	0.630009002	0.071554917	0.23389782	-0.031045507	-0.876265672	0.006740013	-0.401124323	0.660001077
9	0.050978352	-0.301490177	-0.073655947	-0.17853513	0.381753137	0.65355258	0.009583627	-0.097221801	-0.201883987	-0.367705103	-0.146928118	0.304568959	-0.213105161	-0.157121949	0.073597677	-0.00060273	0.197321874	0.472194975	0.619206787
10	0.030955689	0.023065406	-0.301310039	0.115399375	0.374959032	-0.345264571	0.262997304	-0.052536588	0.086325326	-0.490112576	-0.302001683	0.306946188	0.105279479	-0.145389617	-0.050894823	-0.557045929	-0.53671704	-0.421555599	0.572875611
11	0.048195622	-0.402063851	-0.108717131	-0.642916956	0.066889368	-0.143130016	0.064936501	0.153654806	0.248686365	0.145222435	-0.027454568	0.502929249	0.25565816	0.19599361	-0.033759687	-1.148022501	-0.054030775	-0.018890915	0.374611495
12	0.017802348	0.008546436	-0.32545844	-0.925879267	-0.298335274	-0.731801359	-0.105471138	0.197550625	0.311023421	0.085960467	0.278623676	0.458324804	0.295309976	0.194458728	-0.237796118	-0.47432776	0.297740245	-0.133644141	0.911930529
13	0.075809	0.644274305	0.205267557	-1.118610274	-0.418053708	-0.346345413	0.272600819	0.155179489	0.05080517	-0.204091536	-0.13682799	0.456328282	0.174916148	0.333806334	-0.074951565	-0.383891975	0.048698541	-0.330478824	0.603931642
14	0.111317418	-0.093665918	0.091838059	-0.423086157	0.185338153	-0.340490352	0.128192705	0.156731719	0.148865674	-0.308993182	-0.012945931	0.57481748	0.034461565	0.177028944	0.126941065	-0.612897224	-0.467157622	-0.520069364	0.725236754
15	0.105620678	0.111438635	0.232378933	-1.010974405	-0.14044345	0.236835932	0.29751221	0.157857146	0.374885108	-0.45520331	0.410933693	0.493207649	0.184152079	0.113428486	-0.030305371	-0.879547913	0.381671079	-0.143029961	0.514082468
16	0.138388514	0.036430577	0.012879612	0.358383067	0.166084764	0.192700038	0.196690914	-0.011368133	-0.090485026	-0.192682158	-0.414163553	0.532581632	0.063259895	-0.06423673	-0.276350835	-0.325792288	-0.349485744	0.181753916	0.837174148
17	0.137076232	-0.044930253	0.15975769	0.03073381	0.112059984	0.061842528	0.051503213	0.258190364	0.157215587	0.040431649	-0.491118869	0.57480643	0.104319911	0.220278102	-0.008540336	-0.387999029	-0.087308853	-0.017976705	0.964740299
18	0.086230179	0.154119645	-0.027795988	-0.205788185	0.584228265	-0.073339415	0.110532112	0.125110539	0.210801301	0.364955437	0.042449825	0.680157853	0.132702655	-0.067464054	-0.217992014	-0.868689961	-0.092816	-0.123833273	0.715329067
19	0.07437622	0.086156405	0.204369463	0.122617273	0.267330408	0.063517558	0.255045285	0.059996134	0.025187839	-0.122035456	-0.330742108	0.524171709	0.132781532	0.193879696	-0.279684315	-0.203970917	-0.798501817	0.355843738	0.715995994
20	0.051423827	0.057477288	0.106650377	-0.023867197	-0.029273428	0.462506476	0.284808434	0.142844929	0.352938574	0.478314267	-0.130794207	0.579283686	0.135450796	-0.240166664	-0.031722571	0.387645803	-0.815317909	-0.425433152	0.230419052
21	0.090881634	0.181120012	-0.034304442	-0.368706544	0.348601858	-0.296981418	0.118631235	0.030167001	0.240013333	-0.287363562	0.173056721	0.66809819	0.274989062	-0.322664337	-0.142281966	-0.922212146	-0.252504772	-0.238967736	0.507859735
22	0.066724513	0.349450447	0.006132546	-0.150871274	0.462928261	0.203441096	0.190513897	0.175866334	0.296062268	0.214015712	0.230137831	0.346480508	0.044566651	0.25925473	-0.223851296	-0.846349935	-0.457711366	-0.65105637	0.409180618
23	0.16955596	0.5873697	-0.187477573	-0.651656713	-0.308884261	-0.523537764	-0.042342012	0.180350478	0.159235936	0.044125585	0.353334499	0.409795473	0.050836298	0.399382022	-0.089797119	-0.699735412	0.400345856	0.017506292	0.821455673
24	0.147773618	-0.076850424	-0.702645451	0.381179441	0.041994354	-0.137527148	0.212600454	0.297414862	0.245679937	0.034892483	-0.055275449	0.543690382	0.159753958	0.187474026	-0.515611615	-0.042016104	-0.534207065	0.048359479	0.316287821
25	0.136992755	0.043183121	0.006116018	-0.197457742	0.46479258	-0.078596919	0.099945986	0.205145781	0.238464251	-0.279168769	-0.313724843	0.572778481	0.011756923	0.235882076	0.42980007	-0.429291144	-0.439851722	-0.010902226	0.877986337
26	0.108999542	-0.28363398	-0.157933762	-0.770968262	-0.301446038	-1.065319926	-0.607487249	0.234531528	0.232026941	0.175615892	-0.065029082	0.472060698	0.023448541	0.557061632	0.121209706	-0.2393673	-0.275157801	0.118714027	0.187852219
27	0.052753834	0.466534545	0.069163403	-0.183446799	0.200040277	0.133381491	0.109983756	0.139348817	0.748434709	-0.24950336	0.031923091	0.372311854	-0.217839414	0.147090374	0.479367741	-0.305219266	0.162584502	0.545032963	0.095362619
28	0.324322909	-0.108010857	0.279955813	-0.704264148	0.228160221	0.681008519	0.130830044	0.122712827	0.201717664	-0.262535271	0.222760317	0.359728759	0.460278338	0.084474582	-0.197721037	-0.912771284	0.055888516	0.733877095	0.136352462
29	0.20772132	-0.113094378	-0.148941206	-0.677853986	-0.190820067	-0.260213574	0.299473376	0.174704924	0.190019642	-0.086672254	-0.36168797	0.655081654	-0.076421828	-0.179576418	-0.296316053	-1.178131628	0.412505459	-0.58927715	0.277393328
30	0.02270659	-0.074461257	0.887465136	-1.115725772	-0.018393083	-0.231722721	-0.107518586	-0.075572293	0.275055857	-0.561317222	0.07103367	0.66607131	0.061216241	-0.037149835	0.223025998	-0.552635559	0.06659402	-0.082909708	0.710264881
31	0.109392011	-0.084607637	0.373562743	0.40608758	-0.048916523	-0.019645087	-0.101298077	0.014469446	0.031895505	-0.414113645	-0.631088033	0.517781198	0.165015617	0.018661857	-0.169144147	-0.117994885	-0.155721384	-0.016802872	0.355759416
32	0.072606164	-0.517850303	0.144855632	-0.695054469	0.569377539	0.010327873	0.224186904	-0.028245204	0.261856171	-0.179685382	0.048685525	0.610183848	0.311397738	0.789229306	-0.124862337	-0.174245461	-0.539043859	-0.470819161	0.795644355
33	0.261095923	0.165945673	-0.27111599	-0.955841848	-0.337102756	-0.613379807	0.750014363	0.10814995	0.179510407	-0.155964058	0.05449873	0.63613877	0.158854712	0.291521188	-0.368028079	-0.939822413	0.570204156	-0.56958768	-0.055610698
34	0.070615755	-0.28216369	-0.215456308	-0.902580423	0.05016326	-0.176489194	0.096329978	0.245736085	0.004892599	-0.490296686	0.004012003	0.612846177	-0.003629999	-0.182790971	-0.131558981	-0.510542046	0.30239642	-0.343948011	0.813776014
35	0.235604841	0.104870793	-0.095023975	-0.484456301	0.442674782	-0.180566159	-0.007062272	0.029207463	-0.112927714	-0.156038687	-0.228081063	0.519527622	0.053521232	-0.000111811	0.135825234	-0.983580279	-0.141041487	-0.564212559	-0.086563302
36	0.078003439	0.091037366	0.165570991	-0.963393448	0.243832272	-0.086079331	0.218962062	0.169207896	0.370784256	-0.080021463	0.136340814	0.498576644	0.05504561	0.23750458	0.056849462	-1.366761744	-0.051861079	-0.319703907	0.227687731
37	0.070533503	0.106115068	0.173531225	-0.456639654	-0.138226976	-0.470010973	0.242423688	0.072082211	0.233411644	-0.487598262	0.337628265	0.700829055	0.140930974	0.474000178	0.12797424	-0.759829927	0.214488134	-0.423879662	0.582997451
38	0.14468115	0.202860933	0.193451887	-0.448250466	0.323297872	0.23961319	0.058138945	0.131046097	0.127084302	-0.834410746	-0.548126252	0.39557182	-0.051224603	-0.140131604	-0.029088427	-0.679520899	0.01898994	-0.187601481	0.642174002
39	-0.035738091	0.12546551	0.039648807	-0.132502328	0.290553066	0.193805684	0.040651394	0.04001921	0.434013598	-0.351875607	-0.13353129	0.511389864	0.121793198	-0.887902019	-0.007346619	-0.955910813	-0.434926224	-0.676467552	-0.532200771
40	0.12983981	0.166225016	-0.081413495	-0.252621795	0.239689985	-0.032391761	0.071913188	0.076085325	0.376746035	-0.303888318	-0.106989522	0.606346121	0.181399175	-0.33226162	-0.408449172	-1.089591844	-0.422707539	-0.257671934	-0.385684831
41	-0.010625517	0.281366631	0.169250368	-0.77693792	0.455309887	0.183082174	0.150047952	0.303386236	0.614476113	-0.989257683	0.053718433	0.341498669	-0.220160961	0.250737861	0.203584545	-0.415584722	-0.356805485	-0.111865596	0.861034575
42	0.048922818	-0.152807779	-0.084902482	-0.520070706	0.005156008	-0.19633095	-0.389681108	0.066284394	0.187711088	-0.414953019	-0.090302249	0.568333481	0.256497903	-0.362662349	-0.235142427	0.368370587	0.37743021	-0.370190246	0.659328566
43	-0.075492732	-0.066493406	0.115150128	-0.03922383	0.298704405	-0.36779972	-0.352403018	-0.022063591	0.359869074	0.512424132	0.097522092	0.206445393	-0.354376698	0.054123589	-0.286128814	-0.618628453	0.067697022	1.017476063	0.115112041
44	0.165779305	0.108880442	0.426901167	0.064344692	0.051340679	-0.069590017	-0.498715808	0.039728033	-0.02757608	0.519749345	0.17246004	0.468745603	0.083743075	0.448936567	-0.797728319	-1.001719325	0.56011041	-0.353264538	0.178483825
45	0.119985421	0.099360761	0.051409341	-0.477872467	-0.000349795	-0.402548095	-0.166921617	0.179320446	0.416638466	-0.209212556	0.071725367	0.470276057	0.338148241	0.507082187	0.100569597	-0.869800747	0.207567463	0.008795749	0.060139866
46	0.092879174	-0.576330334	-0.137225756	0.286541957	-0.192175354	-0.502957686	0.944239313	0.106967369	-0.018782464	-0.056908374	0.259602777	0.457800861	0.109296048	-0.541606179	-0.279115694	-0.666649615	0.491043605	-0.367842854	-0.079434056
47	0.121883338	0.132848271	0.108219458	-0.766349686	0.255949465	-0.271085129	0.188414193	0.268443076	0.218069912	-0.092249329	0.200745743	0.296016755	0.231718951	0.331516982	0.203992775	-0.571358131	0.178589994	-0.262610163	0.344138422
48	0.081318286	0.110204927	0.172876545	0.051927642	-0.239540712	0.27875126	0.091557745	0.170990936	0.343438763	0.405006635	-0.264720305	0.605540761	0.137593269	-0.01973047	-0.052393976	-0.346439509	-0.290262757	-0.150122875	0.832058109
49	0.159607283	-0.121531031	-0.025847106	-0.655125169	-0.508939927	-0.186138143	-0.122387619	0.178113081	0.29172183	-0.223279169	0.12258885	0.622494662	0.155667355	-0.012546698	-0.032765202	-0.337863942	0.605979022	-0.120448561	0.4528023
50	0.110544853	-0.008108182	-0.0189462	-0.780750285	-0.130387371	-0.449691133	0.388027289	0.180696547	0.235114135	-0.194038761	0.314243729	0.607349729	0.098260334	0.105658271	-0.06409271	-1.184172375	-0.002861017	-0.606052974	0.012037631

	Right Hemisphere																	
	sd-PPBT	Source: M1						Source: Striatum (STR)						Source: Subthalamic Nucleus (STN)				
Subject	Left Hand	Right M1-M1	Right M1-STR	Right M1-STN	Right M1-Gpi	Right M1-Th	Right M1-cCER	Right STR-M1	Right STR-STR	Right STR-STN	Right STR-Gpi	Right STR-Th	Right STR-cCER	Right STN-M1	Right STN-STR	Right STN-STN	Right STN-Gpi	Right STN-Th
Column 1	Column 2	Column 3	Column 4	Column 5	Column 6	Column 7	Column 8	Column 9	Column 10	Column 11	Column 12	Column 13	Column 14	Column 15	Column 16	Column 17	Column 18	Column 19
1	0	0.39018729	-0.281949354	-0.618540621	-0.4015758	-0.31897266	0.675388161	0.230402637	0.715011313	-0.384049212	-0.034213927	0.259594281	0.094428536	0.029040443	0.072802394	-0.183144441	0.146420386	0.188393551
2	-1	0.626926194	-0.136838225	-0.318012115	-0.1134942	-0.034993952	0.198599275	0.221760865	0.745502963	-0.52286223	-0.032944707	0.169211283	0.120116062	0.049062186	0.098107678	-0.055341725	0.075348675	0.10612216
3	0	0.05599228	-0.098855386	0.048627497	0.053799008	0.051295358	-0.101947285	0.121012908	0.719076221	-0.082682906	-0.051909584	0.218516184	0.123565721	-0.10124869	0.087457754	-0.087176606	0.056537874	-0.012638873
4	-1																	
5	0	0.383921623	-0.276460837	-0.664498004	-0.388369086	-0.155642471	0.827300614	0.136449868	0.719952606	-0.3032022	0.011634391	0.210972478	0.124603	0.075071152	0.109655659	-0.143207814	0.163247138	0.164703435
6	-2	0.34968831	-0.001775937	-0.603865573	0.141797203	-0.236259396	0.221122628	0.1227265	0.704555054	-1.053644862	-0.022765864	-0.291445453	0.071071091	0.016752321	0.018346592	-0.1676733	0.040702933	0.245568465
7	-1	0.855649109	-0.211890084	-0.500317158	-0.06064198	-0.256584457	-0.070242539	0.091358589	0.748091357	-0.046114079	0.088188304	-0.052334816	0.067339668	0.000559094	0.07020541	-0.166880173	0.070279568	0.086320073
8	-1	0.59439797	-0.445208616	-0.501041949	-0.344702891	0.294188609	0.06820154	0.162400487	0.724306253	-0.112366585	0.355124114	0.041451423	0.108149716	0.100128213	0.092044799	-0.166964344	0.029357055	0.054214642
9	0	0.60134587	0.126191067	-0.179591311	0.071922963	-0.272517358	0.004244304	0.225797267	0.715981981	-0.051239027	0.214751091	0.102751759	0.090392199	0.065085766	0.063594813	-0.083412864	-0.003066495	0.052518025
10	-1	0.663737958	0.299452095	-0.726339589	-0.615614799	0.150413808	0.280070095	0.103245852	0.659286793	-0.383149725	-1.36317452	0.211525809	0.068763076	0.067707226	0.033489378	-0.103523715	0.061326374	0.010590283
11	-1	0.440701252	-0.192138218	-0.429374698	-0.043099884	0.023422594	-0.136469383	0.170167324	0.733720064	-0.565344754	-0.139374689	0.34256002	0.06704742	0.155638633	0.214873313	-0.173178971	0.159974196	0.101170764
12	-3	0.414583309	-0.437852497	-0.469152499	0.042872148	-0.290658032	-0.01467754	0.041518801	0.687516389	-0.037989742	-0.268726563	0.487794441	0.14722685	0.084072279	0.159465518	-0.118663465	0.031192588	0.195223701
13	0	0.134149429	-0.320919807	-0.645189954	-1.011796509	-0.322841465	-0.187673554	0.332953626	0.674749759	-0.418717888	-0.022046581	-0.017661414	0.113300251	0.147300898	0.030579069	-0.139391918	-0.124176664	0.124839683
14	-4	0.270562683	-0.24751032	-0.125658272	-0.170809256	-0.577181599	0.048483793	0.080387565	0.72137665	-0.006344188	0.526900336	0.367483943	0.114022911	0.158730299	-0.02992159	-0.127155744	-0.233443113	-0.268128407
15	-1	0.541323614	-0.267840773	-0.717131953	-0.671297142	-0.159351645	0.258654096	0.128854859	0.741677856	-0.120014678	-0.136344734	-0.015264077	0.106036053	0.082872557	0.016466501	-0.157068602	0.060647483	0.114249731
16	0	0.807942474	-0.352641275	-0.841573828	-0.290691606	-0.120650243	0.137422347	-0.063485626	0.687712285	0.260912488	0.329793743	0.064673603	0.101308769	0.123274686	0.05027148	-0.138687753	0.056104046	0.112754914
17	0	0.448242903	-0.178125985	0.208133261	-0.190699045	-0.398334981	-0.195332564	0.055207373	0.716648462	-0.266879257	-0.48867693	-0.009225379	0.075884421	-0.098707893	0.056890846	-0.075648839	-0.060516993	0.004340877
18	0	0.104332635	-0.279289008	-0.956800919	-0.094294553	-0.181075104	0.329362654	0.26309051	0.716933785	-0.27392921	0.259849025	0.249430491	0.149989461	0.016796027	0.223219006	-0.187356827	0.072338226	0.180720278
19	-2	0.692525228	-0.179477074	-0.494715216	-0.114796601	-0.601595625	-0.165027864	-0.12709776	0.723778659	-0.496388264	0.080499047	-0.152533364	0.092914611	0.030052673	-0.002814125	-0.028710808	-0.043370839	0.033605116
20	-1	-0.12483714	0.046081889	0.060203351	0.049994166	0.069524263	0.186095695	-0.101764788	0.615288901	-0.297804511	0.039465368	-0.249312067	0.129211682	0.079591898	-0.020606925	-0.167222903	0.143902986	0.231877711
21	-1	0.274526518	-0.065255181	0.148435435	-0.03026995	-0.089577215	-0.0712093	0.114365918	0.718739653	0.031713146	-0.23390437	-0.06598723	0.123788937	-0.13277797	-0.00842986	-0.077140046	0.107272685	-0.106690221
22	-2	0.501769479	-0.271308157	-0.302523085	-0.505583263	-0.768619236	0.178439718	0.139187355	0.744853564	-0.378282812	-0.193804988	0.235358715	0.069240903	0.074801088	0.075389427	-0.097055919	0.124539806	-0.045447096
23	0	0.60145222	-0.259479193	-0.901326388	-0.461047886	-0.113869073	0.1515555	0.03653963	0.765098678	-0.394264144	0.148937394	-0.012371594	0.082331095	0.07609686	0.091766518	-0.149531509	0.111632848	0.103717024
24	0	0.596083165	-0.172776528	-0.669420743	0.220872256	-0.240041156	0.017427618	0.276946466	0.719816284	-0.278123825	0.21501352	0.017223213	0.075183753	0.134027264	0.136542883	-0.182109836	0.06836201	0.224829144
25	0	0.464764304	-0.179163557	-0.393145985	-0.180005341	-0.265887025	-0.020133206	0.042371528	0.711998386	-0.192137551	0.917327473	0.113340023	0.101017795	0.11464084	-0.05820329	-0.111511734	-0.190954431	0.035389347
26	-1	0.295362007	-0.042901597	0.053626609	-0.168057071	0.099014346	-0.12510756	0.50580525	0.657680653	0.06579167	0.073814076	-0.206540057	0.022256549	0.088356714	-0.172364476	-0.072793122	-0.168041062	0.006690501
27	0	0.059202566	0.281790113	-0.229673393	-0.4797886	0.198016834	0.161254535	-0.298280105	0.671949621	-0.384840857	-0.371042561	0.866346626	-0.025149579	-0.107417369	0.134679036	-0.106925824	0.11052957	0.055052519
28	-1	0.603130419	-0.154274278	-0.628739389	0.247060187	-0.208219757	0.134782767	0.177481292	0.705704222	-0.96476324	0.059355044	0.204108432	0.149362947	0.030142103	0.121323824	-0.160751028	-0.21269512	0.133569513
29	-2	0.61658245	-0.255021376	-0.529524372	-0.227976217	-0.159932621	0.033066044	0.127138656	0.732575088	-0.14891922	-0.115988422	0.312152067	0.08003774	0.051040445	0.141998463	-0.219023266	-0.001613015	0.157775374
30	-1	0.870208937	0.039872821	-0.566842667	-0.398710961	0.144680473	-0.077944347	0.172451978	0.656731642	-1.097371733	-0.965974289	0.720006383	0.083053858	0.012914982	0.189440909	-0.11392221	0.075814547	0.304885597
31	-1	0.599254566	0.141796212	-0.745574185	0.285759338	0.05774037	-0.010776799	0.187588717	0.671115915	-0.770361865	0.8758072	0.247439919	0.102574457	0.146292512	0.154749349	-0.08563771	-0.074781516	0.361287633
32	-1	0.171752385	-0.198328466	-0.549224644	-0.151154534	-0.314849358	0.1017691	0.043707688	0.753907334	-0.458410762	-0.111915977	0.173644118	0.117869035	0.040667841	0.077441277	-0.039774147	-0.03232557	0.060564187
33	-1	0.874048622	-0.137751216	-0.635546162	0.048897576	0.028106401	0.055316941	0.187422084	0.671057049	-0.772252684	0.519593369	-0.299865732	0.069342976	0.112949829	0.108132351	-0.120283635	-0.027826241	0.166027185
34	-1	0.255505033	-0.326490767	-0.79050941	0.423143468	-0.387809345	0.014679281	0.164505048	0.695818757	-0.363386215	-0.194612815	0.264285911	0.120076822	0.019400546	0.133652913	-0.168522217	0.146726077	0.058359071
35	-1	0.196219017	-0.181837157	-0.678015918	-0.049913815	-0.226658999	0.478900206	0.226919698	0.664864398	-0.871055782	-0.791914462	-0.155624928	0.187864161	0.160233396	0.16017993	-0.194600803	0.131034982	0.072896384
36	-2	0.864539788	-0.161338435	-0.243420373	-0.316294919	-0.176555064	-0.187706028	0.082007622	0.721690262	0.220520152	0.241548473	0.245431767	0.084020794	0.0552364	-0.016100016	-0.146136515	-0.033087712	-0.013537752
37	-2	0.157481356	-0.287297651	-0.794650311	-0.133005332	0.176158257	0.299650821	0.233835178	0.713717561	-0.918308114	0.170541857	-0.152170953	0.063084151	0.160561473	0.12852504	-0.14358464	0.095159526	0.081758151
38	0	0.767029727	-0.084013642	-0.330258063	0.272297941	0.231529153	0.226967118	-0.162291439	0.715501573	-0.765107005	0.120597438	0.018078069	0.087712536	0.265341411	0.09047759	-0.206192136	0.084250284	0.062844499
39	-1	0.67309083	-0.042474636	-0.601581486	-0.918674651	0.247557073	0.116930187	0.109199244	0.708341652	-1.015000509	-0.271494931	0.23736742	0.071490766	0.092688053	0.133416738	-0.182771957	0.333490243	0.090887826
40	0	0.595002849	-0.336562511	-0.341219638	-0.312245716	-0.031007775	0.318754359	0.091651034	0.711315974	-0.325288049	-0.034960126	-0.052048732	0.052223924	0.17663316	-0.03388146	-0.096206414	0.008380937	0.165865536
41	-1	0.777345851	-0.136330979	-0.129038304	-0.319464659	-0.143415955	-0.167918631	0.019384625	0.738626075	-0.215256285	-0.042464026	-0.222248842	0.105515276	0.00427243	0.051711956	-0.144047002	0.062104488	-0.003694224
42	-1	0.637116494	0.191124145	-0.637580505	-0.243554313	-0.06368194	0.155942969	0.327780379	0.658953955	-0.832504381	-0.140663293	0.121779658	0.113920332	0.096820322	0.076495282	-0.061227014	0.031557017	-0.0349498
43	-1	0.467881509	-0.134533979	-0.812297147	-0.250050788	0.166107039	0.593563532	0.220741766	0.657375946	-0.789006894	0.31707137	-0.338374989	0.079771693	0.103085366	0.112957525	-0.047853808	0.11249278	0.037555439
44	-1	0.226465507	-0.20897288	-0.496091314	0.078109669	-0.561847364	0.48362275	0.340998607	0.730424079	-0.570295368	0.134359467	-0.190560593	0.091062183	0.10116326	0.102861237	-0.117606109	-0.000859827	0.04572254
45	0	0.402386866	-0.211826045	-0.441529955	0.011467159	-0.281504774	0.520351775	0.2577626	0.684811027	-0.485170459	-0.00781413	0.461133211	0.192942415	-0.000980188	0.135300056	-0.164310403	0.068047085	0.149415616
46	0	0.270086066	-0.02007527	0.263870027	0.323799516	-0.127890188	0.553453097	0.171910618	0.703680941	0.099834893	0.623099408	0.113817495	0.140728831	-0.048420789	0.053856774	-0.132855806	0.067248951	0.02227738
47	-1	0.538718116	-0.125386114	-0.418120771	-0.029182544	-0.25553983	0.267840997	-0.008628029	0.671601412	-0.943203877	0.11101942	-0.446164885	0.077243764	0.115579832	0.041886503	-0.18157801	0.145829344	0.250097621
48	0	0.641133261	-0.149414036	-0.525850373	-0.587194895	0.227682839	0.345595023	-0.182019059	0.656863756	-0.321945115	-0.255210527	0.011922689	0.075223173	0.106464789	0.141813042	-0.094616491	-0.008276454	0.227882719
49	0	0.495063201	0.288614377	-0.931126859	0.245681323	0.16024788	-0.105246343	0.253493248	0.660762166	-1.047280954	0.024708433	0.395970698	0.019123502	0.138426616	0.142598077	-0.135124767	0.099742352	0.187969168
50	0	0.384363226	-0.10508306	-0.25088344	-0.145310026	-0.379825587	0.229698175	0.341873788	0.707343615	-0.205958487	0.178578887	0.026112761	0.157594027	0.010303201	0.145254406	-0.192056152	0.131571046	0.245604226

	Right Hemisphere																		
	Source: Subthalamic Nucleus (STN)	Source: Globus Pallidus Internus (Gpi)						Source: Thalamus (Th)						Source: Contralateral Cerebellum (cCER)					
Subject	Right STN-cCER	Right Gpi-M1	Right Gpi-STR	Right Gpi-STN	Right Gpi-Gpi	Right Gpi-Th	Right Gpi-cCER	Right Th-M1	Right Th-STR	Right Th-STN	Right Th-Gpi	Right Th-Th	Right Th-cCER	Right cCER-M1	Right cCER-STR	Right cCER-STN	Right cCER-Gpi	Right cCER-Th	Right cCER-cCER
Column 1	Column 20	Column 21	Column 22	Column 23	Column 24	Column 25	Column 26	Column 27	Column 28	Column 29	Column 30	Column 31	Column 32	Column 33	Column 34	Column 35	Column 36	Column 37	Column 38
1	0.089327045	0.24982909	0.015083396	-0.118219291	0.348113797	0.041929991	0.158803976	0.313214624	0.316383549	0.186255058	0.09840272	0.535161277	0.232538517	0.305043771	-0.483593048	-0.727761295	-0.449874398	-0.702332877	0.069255809
2	0.108360172	0.101344128	0.0313117	-0.57690065	0.352127643	-0.073300913	0.004128072	0.020453978	-0.161534107	-1.049388016	-0.211407347	0.516933917	0.221035882	0.008342482	-0.375144882	-0.725153392	-0.205365057	0.148427782	0.640974265
3	0.190072325	-0.018128645	0.06082791	0.074872334	0.36237137	0.06425214	0.087876628	0.308817793	0.158927091	0.01382293	0.133054447	0.54689382	0.102379753	-0.112995404	0.282940281	-0.112819608	-0.033861687	-0.014009666	0.50678538
4																			
5	0.100421913	0.038185306	0.106787552	-0.247240034	0.353847472	0.161750273	0.088403387	0.349829455	0.104604995	0.043433951	-0.075876123	0.514608549	0.464013886	0.128346284	0.008092163	-0.972757938	-0.512557049	-0.453117985	-0.897747375
6	0.101329617	0.056741109	0.187365045	-0.992778682	0.33285502	-0.30387291	0.084388059	0.162046824	0.126263853	-0.400920446	0.227484475	0.548451133	0.21590447	-0.091436973	0.173973159	-0.935815085	0.369341481	-0.29595399	0.787617346
7	0.16358898	-0.088564444	-0.115057511	-0.211007902	0.250476264	-0.03794265	0.15196587	0.065523896	-0.22017842	-0.478213859	-0.083120442	0.572702492	0.185134791	-0.181369003	-0.559561682	-0.964999315	-0.296895804	-0.187796596	0.368942487
8	0.206214332	0.131923726	0.088503675	-0.020572293	0.363805752	0.092310988	0.141425232	0.043327042	-0.499965749	-0.681013825	-0.569357437	0.535411206	-0.012564213	0.037264225	-0.283264974	-0.67838647	-0.116973432	-0.074920966	1.044350231
9	0.177975495	-0.06185181	-0.129346759	-0.348290907	0.295615478	-0.02175966	-0.069650389	-0.042237021	-0.182857416	-0.190707602	-0.147509234	0.566423057	0.155903142	0.049940569	0.080908676	-0.537249732	-0.158641316	-0.142896075	0.907535537
10	0.119986365	0.197428624	0.070884888	0.040335599	0.302180803	0.126940567	0.15250277	-0.047288409	0.393041928	-0.272627425	-0.63040309	0.557796611	-0.195915834	0.234876538	0.331345223	-1.217286345	-0.46116605	0.108064049	0.642685204
11	0.18261892	0.116636556	0.094437968	-0.198969091	0.282894135	0.220419535	0.040717698	0.097256123	-0.100417996	-0.698476813	-0.316800265	0.524510626	-0.38080567	-0.045433052	-0.004357253	-0.451587199	0.088519443	-0.100284538	0.996022986
12	0.165905402	-0.019218277	-0.370116382	-0.594495244	0.262430236	-0.297654823	-0.089376592	0.179548769	0.047353574	0.076478926	0.578856372	0.541616125	0.29102333	0.15092344	-0.135635398	-0.586981965	-0.076531783	-0.043400912	1.067235205
13	-0.076932001	-0.090057113	0.359315828	0.143838855	0.286293845	0.267625435	0.185036985	0.462824298	0.277337597	0.263959963	-0.215769185	0.530151334	-0.026215615	0.260830319	-0.157139903	0.124959496	-0.604580873	0.075948204	0.526646617
14	0.187292513	0.272263892	-0.15059455	0.83991856	0.306973784	-0.702972053	0.046987692	-0.08433703	0.642219323	-0.909446882	0.387441608	0.494459542	0.163775279	-0.013759134	-0.067143342	-0.316928227	-0.071407155	-0.251635827	1.020395858
15	0.073000493	0.07746765	0.030938614	0.023926052	0.363603955	-0.109726862	0.070615372	0.129753164	0.068845088	-0.563470231	-0.212429532	0.564051536	0.162277389	0.068651106	-0.540038807	-0.706413844	-0.689834543	-0.536550837	0.50435406
16	0.142675871	-0.004466895	-0.007044241	-0.383704585	0.379373701	0.020949715	0.168604671	0.047885178	0.235631337	-0.514402759	0.010312021	0.565359635	0.227713894	-0.028594996	-0.198068	-1.035436565	-0.274875645	-0.025704374	0.677525015
17	0.069211844	0.14212671	0.047339025	-0.066164134	0.344760183	0.048993796	0.044833812	0.044774825	-0.026617279	-0.772402148	-0.069048075	0.544346476	0.16735912	-0.085955084	-0.319161082	-0.638985845	-0.205997433	-0.242621632	0.909768836
18	0.046464134	0.295649577	-0.016557262	-0.419187662	0.333459657	0.031274894	0.295252091	0.266826278	0.246725126	-0.113214814	0.081062518	0.5539454	0.432592985	0.464803242	-0.264427224	-1.243101929	-0.033351797	-0.144294081	0.244481961
19	0.149901652	-0.274488276	0.074890429	-0.242061149	0.420417254	0.014294595	0.070718627	0.041357477	-0.105490505	-0.439342178	-0.270943324	0.49860571	0.406741442	0.062689268	-0.40398537	-1.009919651	-0.564229004	0.319728361	0.657923211
20	0.185325785	-0.31261837	0.349720767	-0.286819725	0.355380933	-0.294571093	0.130632094	0.124125133	0.391800666	-0.251428011	0.2049063	0.532924137	0.141588001	-0.426690974	0.374568226	-0.915183726	0.372376343	-0.344076877	0.307423618
21	0.066278276	-0.174376814	0.024751802	-0.41199848	0.277818884	0.031390195	0.207622737	0.019220174	0.414492368	0.408172028	-0.041337808	0.55270174	0.200338021	-0.216310145	-0.152126024	-0.626438626	-0.046176806	-0.312670194	0.603936523
22	0.163807529	0.150732835	0.019753016	-0.309795928	0.367504689	-0.009268665	-0.005523173	0.10981851	0.076746419	0.074922797	0.032456239	0.51387232	-0.01916998	-0.137371338	-0.450150591	-0.26251155	-0.27750034	-0.745612925	0.807679189
23	0.210440846	-0.015526191	-0.068880734	-0.074925074	0.292131236	0.023597252	0.156634712	0.189768088	-0.107270015	-0.900447716	-0.304364047	0.566824776	0.119818201	0.272381963	-0.415415708	-0.935315196	-0.563195344	0.007299173	0.7525876
24	0.068195425	-0.003817422	-0.159628206	-1.045514697	0.333113388	-0.265430567	0.084473687	0.158963994	0.156996018	0.032721775	0.182026672	0.557117527	0.05476723	-0.005440568	-0.388944749	-0.696470708	0.041765162	-0.468651694	0.597164684
25	0.241194428	-0.043541086	0.222845129	0.127410042	0.266855781	0.125737266	-0.041429916	0.081376369	-0.108922826	0.078530199	-0.146247609	0.575144286	0.211659344	0.153448348	-0.661077675	-0.08737841	-0.959579265	-0.322457627	0.649499132
26	0.072351693	0.179802683	0.253835649	0.15837478	0.406239326	-0.034654715	0.011747002	-0.201201409	-0.27841309	-0.484971355	-0.13420366	0.505680244	0.37984438	0.051237878	-0.053712697	-0.023885824	-0.431927181	-0.414295303	0.135305794
27	0.248390932	0.261258775	-0.227003696	-0.472615647	0.26776627	0.218472652	0.090862516	-0.007828955	0.949488583	-0.663024695	-0.032257381	0.508993969	-0.630092964	-0.012502854	0.818869422	-0.409055446	-0.341072821	0.638580319	0.419212197
28	0.028081042	0.113723423	-0.169545579	-0.664918109	0.246316326	-0.361108978	-0.045920032	0.10975388	0.296534636	-0.571541015	0.090998479	0.537120233	0.390945225	0.174780816	-0.092597769	-0.810659638	0.234882719	-0.01889172	0.333791314
29	0.103595977	0.055155777	-0.200026426	-0.440760601	0.395140989	0.007668717	0.010410564	0.220290245	-0.33464344	-0.596034475	0.090919573	0.54691208	0.249074914	0.014947137	-0.559708861	-0.888337293	0.087487273	-0.121994296	0.624449506
30	0.009437889	-0.0036957	0.026404928	0.157586252	0.356916064	-0.043171002	0.038361421	0.133336292	0.96722745	-1.358480811	-0.518028061	0.511580132	-0.01267231	-0.104139926	0.075662635	-0.788666079	-0.371643063	0.138745581	1.053767438
31	0.320022462	-0.094556265	0.033219255	0.077658987	0.299524699	0.030284119	0.097248302	0.171563091	0.214747945	-0.636032432	-0.051743538	0.530496178	0.674686264	0.065014094	-0.064455612	-0.840740044	-0.262499219	0.732104335	-0.003343687
32	0.068474886	-0.023971563	0.021937937	-0.248539542	0.424867852	-0.310512603	0.119782441	-0.045917155	-0.04474858	-0.648995286	-0.105385311	0.528836431	0.076490887	-0.280682713	-0.11403054	-0.530511082	-0.210213037	0.013289618	0.819634054
33	0.096591203	-0.020171511	-0.135846508	-0.762502085	0.274601338	-0.381567948	0.112275929	0.033204406	0.142998223	-0.336549183	0.211464624	0.530308493	0.236684509	-0.094173723	-0.338747382	-0.884957204	0.541527756	-0.600211033	0.626604426
34	0.18625607	0.014447952	-0.144508104	-0.714313256	0.290120395	-0.330327616	-0.021010218	0.16307417	0.068127109	0.192778249	-0.123236701	0.574778531	0.446566173	0.437490304	-0.079315195	-0.822938788	0.404827328	-0.441099664	0.810691614
35	0.095888433	-0.006871838	0.007804687	-0.44764919	0.362628079	-0.168629537	0.005558794	0.052261779	0.053953061	-0.210175984	-0.060583301	0.586962575	0.28454235	0.0587019	-0.307287294	-1.103610033	-0.314564209	-0.341362244	0.168923573
36	0.132223917	0.071392572	-0.000291867	-0.111326729	0.317963846	-0.017349293	0.095132188	-0.003732804	0.133065415	-0.105163252	0.114155258	0.577382773	0.213307644	-0.251259847	-0.541139053	-0.555785992	-0.408387163	-0.37481162	0.726456587
37	0.09353034	0.317932822	0.0855134	-0.736056684	0.348892999	-0.32675663	0.038287977	-0.089976155	-0.073098972	-0.745367847	-0.123021504	0.506586159	0.294214805	-0.170279409	-0.280393834	-1.051198416	-0.141895113	0.422181622	0.207563123
38	0.236847738	-0.188433271	0.166797835	-1.185310296	0.302740085	-0.128760429	0.094063102	-0.116930285	0.146316202	-1.067121396	0.0683721	0.500535042	-0.249514056	-0.133529899	0.308720476	-0.72290767	0.353278421	0.114403419	0.646282795
39	0.149487304	0.100315751	0.267866659	0.25679371	0.256717154	0.023883026	0.081334601	0.133267176	0.017284936	-0.667662299	-0.747692553	0.55562598	0.025138566	0.196534055	0.002941264	-0.639918808	-0.670702917	0.269973108	0.907095412
40	0.213198459	0.044719973	0.087065911	-0.020915906	0.388486519	0.09272479	0.042047467	0.035532627	-0.186005762	-0.639543909	-0.110482493	0.542988969	0.142525247	-0.462932817	-0.445667742	-0.664578182	-0.422603148	-0.189949712	-0.146158063
41	0.197889059	0.032835277	-0.070088679	-0.479535915	0.321163779	-0.331377589	0.231402759	-0.02703016	0.160111493	0.329482943	0.095864173	0.564753738	-0.135821554	-0.106011933	-0.346337102	-0.99844414	0.015542798	-0.780190675	0.048469086
42	-0.005756136	-0.02396362	-0.065904846	-0.418671225	0.391837658	-0.105833824	-0.012670645	0.085961504	0.178739347	0.006922243	-0.055871897	0.563359912	0.364772936	-0.291048463	-0.417494084	-0.446635962	-0.192179087	-0.432298805	0.678382476
43	0.060266397	0.093846329	-0.121082495	-0.442440091	0.443601697	0.179972366	0.195713362	0.061490172	-0.311667209	-0.640040587	-0.530201667	0.518151422	0.098821782	-0.241291564	-0.676595137	-1.169544589	-0.299368975	0.118898219	0.291588437
44	0.234629494	0.1694432	-0.12460568	-0.239339423	0.274345545	-0.218603305	0.190707431	0.227821901	0.172653036	-0.044490295	-0.007141395	0.577485162	-0.202289581	-0.135547204	-0.327770105	-0.995406907	-0.075918596	-0.631814066	-0.011290456
45	0.11738488	0.059459462	-0.207949196	-0.456740254	0.348501883	0.010295307	0.129597182	0.144638363	0.339866448	-0.221035871	0.101861112	0.509954507	0.507768972	0.207082897	-0.156194993	-1.020683455	0.077660105	-0.154185502	-0.838896002
46	0.322974335	-0.281205751	-0.208926715	0.398822178	0.275881584	-0.168456432	0.485506785	0.200229092	0.119177476	-0.043992218	0.065244562	0.536285125	0.206644052	-0.484193983	-0.473383592	-0.150547058	0.518619667	-0.687417563	-0.957888833
47	0.217087461	0.05180775	0.173906116	-0.436956406	0.266472269	-0.261478661	0.069521334	0.070773263	0.050708041	0.003353011	-0.001190646	0.547645365	0.339785409	-0.048169304	0.452555185	-0.604014177	0.374166019	-0.456658002	0.800903413
48	0.314683682	0.108886237	-0.189679752	-0.336478944	0.420446271	-0.045140553	0.126104281	0.018604144	0.680663105	-0.814550373	-0.194776455	0.510360994	0.297022201	0.187360912	-0.295281314	-1.136498824	-0.576634543	0.694163671	0.240207582
49	0.315047822	0.107992798	0.32642617	-0.318331948	0.250323551	0.15347884	-0.045481665	0.173977975	0.606543209	-0.205278242	-0.607051717	0.536527669	0.146633753	0.340646579	0.057741108	-0.725728955	0.037168798	-0.106302719	0.775832868
50	0.093603553	0.15864244	0.104760323	-0.416557312	0.384580642	0.047774326	0.104084542	0.073834012	0.249894997	0.455081685	-0.039117648	0.527639439	0.210603779	0.15901883	-0.014308244	-1.238048943	-0.024542847	-0.542819098	0.419842104

Subject 4's right hemisphere estimates could not be estimated due to the subject's right STN being not identifiable.
