# Supplementary material for: Motor network dynamic resting state fMRI connectivity of neurotypical children in regions affected by cerebral palsy
Source: Front Hum Neurosci. 2024 May 21;18:1339324. doi: 10.3389/fnhum.2024.1339324 (PMC11148452; doi:10.3389/fnhum.2024.1339324)
Supplement: Supplementary file 2 [file Table_2.pdf]

Supplementary Table S2a. Left Hemisphere Connectivity Parameter Estimates and Confidence Intervals

		Source											
		L M1		L STR		L STN		L GPi		L THAL		R CER	
		M	(95% CI)	M	(95% CI)	M	(95% CI)	M	(95% CI)	M	(95% CI)	M	(95% CI)
Sink	**L M1**	0.47	(0.44 , 0.50)	0.13	(0.09 , 0.16)	0.06	(0.05 , 0.08)	0.05	(0.02 , 0.09)	0.13	(0.10 , 0.16)	0.09	(0.05 , 0.14)
	**L STR**	0.00	(0.00 , 0.00)	0.69	(0.65 , 0.72)	0.10	(0.08 , 0.12)	0.00	(0.00 , 0.00)	0.18	(0.14 , 0.21)	-0.07	(-0.12 , -0.03)
	**L STN**	-0.45	(-0.51 , -0.40)	-0.43	(-0.48 , -0.38)	-0.10	(-0.13 , -0.07)	-0.35	(-0.40 , -0.29)	-0.15	(-0.20 , -0.11)	-0.54	(-0.59 , -0.48)
	**L GPi**	0.00	(0.00 , 0.00)	0.10	(0.04 , 0.16)	0.03*	(-0.01 , 0.07)	0.07	(0.02 , 0.12)	0.00	(0.00 , 0.00)	-0.03*	(-0.09 , 0.03)
	**L THAL**	-0.17	(-0.22 , -0.12)	-0.07	(-0.12 , -0.02)	0.07	(0.05 , 0.09)	-0.13	(-0.18 , -0.09)	0.50	(0.47 , 0.54)	-0.14	(-0.20 , -0.09)
	**R CER**	0.03*	(-0.02 , 0.09)	0.10	(0.05 , 0.15)	0.10	(0.08 , 0.11)	0.08	(0.05 , 0.12)	0.10	(0.07 , 0.13)	0.41	(0.36 , 0.46)

Parameter estimates for the Left Hemisphere model. All estimates are listed in Hz with the exception of self-connections. The self-connections are represented in the log scaling factor they are converted to during DCM inversion. This transformation ensures that all self-connections are negative when expressed in Hz, thus lending stability to the model. As such, more positive parameter estimates on self-connections indicate more self-inhibition and more negative (or less positive) parameter estimated on self-connections indicate less self-inhibition. The output from SPM reports self-connection parameter estimates in the log-scale, value, not Hz. (Note that, in the matrices in Fig. 1A and 1B, the diagonal has been converted to Hz using the formula $A_{Hz} = -0.5 \times \exp(A)$ where A is the log-scaled parameter estimate value).

All non-zero parameter estimates had a posterior probability of 0.95 or greater with the exception of those with an asterisk. The M1→ R Cerebellum had a posterior probability of 0.66, the STN→GPi connection had a posterior probability of 0.75, and the Right Cerebellum→GPi connection had a posterior probability of 0.54.

Supplementary Table S2b. Right Hemisphere Connectivity Parameter Estimates and Confidence Intervals

		Source											
		L M1		L STR		L STN		L GPi		L THAL		R CER	
		M	(95% CI)	M	(95% CI)	M	(95% CI)	M	(95% CI)	M	(95% CI)	M	(95% CI)
Sink	**L M1**	0.45	(0.41 , 0.49)	0.13	(0.10 , 0.17)	0.07	(0.05 , 0.08)	0.00	(0.00 , 0.00)	0.10	(0.07 , 0.13)	0.00	(0.00 , 0.00)
	**L STR**	-0.13	(-0.17 , -0.10)	0.69	(0.66 , 0.72)	0.08	(0.06 , 0.10)	0.00	(0.00 , 0.00)	0.12	(0.07 , 0.16)	-0.15	(-0.20 , -0.10)
	**L STN**	-0.43	(-0.47 , -0.38)	-0.38	(-0.43 , -0.32)	-0.14	(-0.16 , -0.11)	-0.26	(-0.31 , -0.21)	-0.31	(-0.37 , -0.26)	-0.66	(-0.71 , -0.61)
	**L GPi**	-0.13	(-0.18 , -0.09)	0.00	(0.00 , 0.00)	0.05	(0.03 , 0.07)	0.32	(0.29 , 0.35)	-0.09	(-0.13 , -0.05)	-0.15	(-0.20 , -0.10)
	**L THAL**	-0.13	(-0.17 , -0.09)	0.10	(0.05 , 0.14)	0.09	(0.07 , 0.11)	-0.05	(-0.08 , -0.03)	0.54	(0.50 , 0.57)	-0.15	(-0.20 , -0.10)
	**R CER**	0.13	(0.09 , 0.17)	0.09	(0.06 , 0.12)	0.13	(0.12 , 0.15)	0.09	(0.06 , 0.11)	0.16	(0.11 , 0.20)	0.44	(0.38 , 0.49)

Parameter estimates for the Right Hemisphere model. All estimates are listed in Hz with the exception of self-connections. The self-connections are represented in the log scaling factor they are converted to during DCM inversion. This transformation ensures that all self-connections are negative when expressed in Hz, thus lending stability to the model. As such, more positive parameter estimates on self-connections indicate more self-inhibition and more negative (or less positive) parameter estimated on self-connections indicate less self-inhibition. The output from SPM reports self-connection parameter estimates in the log-scale, value, not Hz. (Note that, in the matrices in Fig. 1A and 1B, the diagonal has been converted to Hz using the formula $A_{Hz} = -0.5 \times \exp(A)$ where A is the log-scaled parameter estimate value).

There were no non-zero parameter estimates had a posterior probability that was less than 0.95.
