# Supplementary material for: Motor network dynamic resting state fMRI connectivity of neurotypical children in regions affected by cerebral palsy
Source: Front Hum Neurosci. 2024 May 21;18:1339324. doi: 10.3389/fnhum.2024.1339324 (PMC11148452; doi:10.3389/fnhum.2024.1339324)
Supplement: Supplementary file 3 [file Table_3.pdf]

Supplementary Table S3a. Left Hemisphere Connectivity association with age parameter estimates and confidence intervals.

		Source											
		L M1		L STR		L STN		L GPi		L THAL		R CER	
		M	(95% CI)	M	(95% CI)	M	(95% CI)	M	(95% CI)	M	(95% CI)	M	(95% CI)
Sink	L M1	0.00	(0.00 , 0.00)	0.00	(0.00 , 0.00)	0.00	(0.00 , 0.00)	0.00	(0.00 , 0.00)	0.00	(0.00 , 0.00)	-0.03	(-0.05 , -0.02)
	L STR	0.03	(0.02 , 0.04)	-0.02	(-0.03 , -0.01)	0.00	(0.00 , 0.00)	0.00	(0.00 , 0.00)	0.03	(0.02 , 0.04)	0.00	(0.00 , 0.00)
	L STN	0.00	(0.00 , 0.00)	0.00	(0.00 , 0.00)	0.00	(0.00 , 0.00)	0.00	(0.00 , 0.00)	0.00	(0.00 , 0.00)	0.00	(0.00 , 0.00)
	L GPi	0.00	(0.00 , 0.00)	0.00	(0.00 , 0.00)	0.00	(0.00 , 0.00)	0.00	(0.00 , 0.00)	0.00	(0.00 , 0.00)	0.06	(0.04 , 0.08)
	L THAL	0.03	(0.01 , 0.05)	0.00	(0.00 , 0.00)	0.00	(0.00 , 0.00)	0.00	(0.00 , 0.00)	0.00	(0.00 , 0.00)	0.00	(0.00 , 0.00)
	R CER	0.03	(0.01 , 0.04)	-0.04	(-0.06 , -0.02)	0.00	(0.00 , 0.00)	0.00	(0.00 , 0.00)	-0.01*	(-0.02 , 0.01)	-0.06	(-0.08 , -0.04)

Parameter estimates for the Left Hemisphere associated with age. For self-connections, a positive parameter estimate on a self-connection (positive effect of a covariate) indicates a positive relationship between the covariate and the level of self-inhibition and a negative parameter estimate on a self-connection (negative effect of a covariate) indicates a negative relationship between the covariate and the level of self-inhibition.

For all other connections, positive parameters indicate that more excitatory/less inhibitory connectivity values are associated with higher age (positive relationship) and negative parameter values indicate that more inhibitory/less excitatory connectivity values are associated with higher age (negative relationship).

All non-zero parameter estimates had a posterior probability of 0.95 or greater with the exception of those with an asterisk. The posterior probabilities of Thalamus→R Cerebellum is 0.5.

Supplementary Table S3b. Right Hemisphere Connectivity association with age parameter estimates and confidence intervals

		Source											
		L M1		L STR		L STN		L GPi		L THAL		R CER	
		M	(95% CI)	M	(95% CI)	M	(95% CI)	M	(95% CI)	M	(95% CI)	M	(95% CI)
Sink	L M1	0.00	(0.00 , 0.00)	0.00	(0.00 , 0.00)	0.01*	(0.00 , 0.02)	0.00	(0.00 , 0.00)	-0.04	(-0.05 , -0.03)	-0.03	(-0.04 , -0.01)
	L STR	0.01*	(0.00 , 0.03)	-0.04	(-0.05 , -0.03)	0.00	(0.00 , 0.00)	-0.02	(-0.03 , -0.01)	0.01*	(-0.01 , 0.03)	0.00	(0.00 , 0.00)
	L STN	0.00	(0.00 , 0.00)	-0.04	(-0.06 , -0.02)	0.00	(0.00 , 0.00)	0.00	(0.00 , 0.00)	0.00	(0.00 , 0.00)	-0.03	(-0.05 , -0.01)
	L GPi	0.01*	(-0.01 , 0.03)	0.03	(0.01 , 0.05)	0.00	(0.00 , 0.00)	0.00	(0.00 , 0.00)	0.00	(0.00 , 0.00)	0.03	(0.01 , 0.05)
	L THAL	0.03	(0.01 , 0.05)	-0.01*	(-0.03 , 0.01)	0.00	(0.00 , 0.00)	-0.02	(-0.03 , -0.01)	0.00	(0.00 , 0.00)	0.00	(0.00 , 0.00)
	R CER	0.00	(0.00 , 0.00)	0.00	(0.00 , 0.00)	0.01	(0.01 , 0.02)	0.00	(0.00 , 0.00)	0.00	(0.00 , 0.00)	-0.05	(-0.07 , -0.02)

Parameter estimates for the Right Hemisphere associated with age. For self-connections, a positive parameter estimate on a self-connection (positive effect of a covariate) indicates a positive relationship between the covariate and the level of self-inhibition and a negative parameter estimate on a self-connection (negative effect of a covariate) indicates a negative relationship between the covariate and the level of self-inhibition.

For all other connections, positive parameters indicate that more excitatory/less inhibitory connectivity values are associated with higher age (positive relationship) and negative parameter values indicate that more inhibitory/less excitatory connectivity values are associated with higher age (negative relationship).

All non-zero parameter estimates had a posterior probability of 0.95 or greater with the exception of those with an asterisk. The posterior probabilities of the asterisked values are: M1→STR: 0.72; M1→Gpi: 0.61, STR→THAL: 0.6, STN→M1: 0.71, THAL→STR: 0.52.
