# Supplementary material for: Motor network dynamic resting state fMRI connectivity of neurotypical children in regions affected by cerebral palsy
Source: Front Hum Neurosci. 2024 May 21;18:1339324. doi: 10.3389/fnhum.2024.1339324 (PMC11148452; doi:10.3389/fnhum.2024.1339324)
Supplement: Supplementary file 4 [file Table_4.pdf]

Supplementary Table S4a. Left Hemisphere Connectivity association with (right hand) Purdue Pegboard Test (transformed) score parameter estimates and confidence intervals.

		Source											
		L M1		L STR		L STN		L GPi		L THAL		R CER	
		M	(95% CI)	M	(95% CI)	M	(95% CI)	M	(95% CI)	M	(95% CI)	M	(95% CI)
Sink	L M1	0.00	(0.00 , 0.00)	0.00	(0.00 , 0.00)	0.00	(0.00 , 0.00)	0.12	(0.08 , 0.16)	0.00	(0.00 , 0.00)	-0.09	(-0.15 , -0.04)
	L STR	0.00	(0.00 , 0.00)	-0.17	(-0.21 , -0.12)	0.00	(0.00 , 0.00)	0.00	(0.00 , 0.00)	0.07	(0.03 , 0.11)	0.00	(0.00 , 0.00)
	L STN	0.10	(0.03 , 0.16)	0.09	(0.03 , 0.16)	-0.09	(-0.12 , -0.05)	0.12	(0.05 , 0.18)	0.09	(0.04 , 0.15)	0.00	(0.00 , 0.00)
	L GPi	0.04*	(-0.04 , 0.12)	0.00	(0.00 , 0.00)	0.00	(0.00 , 0.00)	-0.05*	(-0.12 , 0.03)	0.04*	(-0.03 , 0.10)	0.15	(0.09 , 0.21)
	L THAL	0.00	(0.00 , 0.00)	0.00	(0.00 , 0.00)	0.00	(0.00 , 0.00)	0.00	(0.00 , 0.00)	-0.04*	(-0.09 , 0.01)	0.15	(0.09 , 0.22)
	R CER	0.10	(0.05 , 0.15)	-0.12	(-0.17 , -0.06)	0.00	(0.00 , 0.00)	0.00	(0.00 , 0.00)	-0.08	(-0.12 , -0.04)	0.00	(0.00 , 0.00)

Parameter estimates for the Left Hemisphere associated with right hand Purdue Pegboard Test (PPBT) score. For self-connections, a positive parameter estimate on a self-connection (positive effect of a covariate) indicates a positive relationship between the covariate and the level of self-inhibition and a negative parameter estimate on a self-connection (negative effect of a covariate) indicates a negative relationship between the covariate and the level of self-inhibition.

For all other connections, positive parameters indicate that more excitatory/less inhibitory connectivity values are associated with higher PPBT scores (positive relationship), and negative parameter values indicate that more inhibitory/less excitatory connectivity values are associated with higher PPBT scores (negative relationship).

All non-zero parameter estimates had a posterior probability of 0.95 or greater with the exception of those with an asterisk. The posterior probabilities of the asterisked values are: M1→GPi: 0.54, GPi→GPi: 0.66, THAL→GPi: 0.61, THAL→THAL: 0.71

Supplementary Table S4b. Right Hemisphere Connectivity association with (left hand) Purdue Pegboard Test score parameter estimates and confidence intervals

		Origin											
		L M1		L STR		L STN		L GPi		L THAL		R CER	
		M	(95% CI)	M	(95% CI)	M	(95% CI)	M	(95% CI)	M	(95% CI)	M	(95% CI)
Target	L M1	0.00	(0.00 , 0.00)	0.00	(0.00 , 0.00)	0.00	(0.00 , 0.00)	0.00	(0.00 , 0.00)	0.10	(0.07 , 0.13)	0.03*	(-0.03 , 0.08)
	L STR	0.06	(0.02 , 0.10)	0.00	(0.00 , 0.00)	0.01*	(-0.01 , 0.04)	0.06	(0.03 , 0.10)	0.00	(0.00 , 0.00)	0.00	(0.00 , 0.00)
	L STN	0.00	(0.00 , 0.00)	0.00	(0.00 , 0.00)	0.00	(0.00 , 0.00)	0.00	(0.00 , 0.00)	0.03*	(-0.04 , 0.10)	0.00	(0.00 , 0.00)
	L GPi	0.00	(0.00 , 0.00)	0.00	(0.00 , 0.00)	0.04	(0.02 , 0.07)	0.00	(0.00 , 0.00)	-0.15	(-0.19 , -0.10)	-0.08	(-0.14 , -0.03)
	L THAL	0.07	(0.03 , 0.12)	0.00	(0.00 , 0.00)	0.06	(0.03 , 0.08)	0.23	(0.19 , 0.26)	0.08	(0.04 , 0.12)	0.00	(0.00 , 0.00)
	R CER	0.08	(0.03 , 0.13)	0.00	(0.00 , 0.00)	0.00	(0.00 , 0.00)	0.07	(0.04 , 0.10)	0.00	(0.00 , 0.00)	-0.19	(-0.25 , -0.12)

Parameter estimates for the Right Hemisphere associated with left hand Purdue Pegboard Test (PPBT) score. For self-connections, a positive parameter estimate on a self-connection (positive effect of a covariate) indicates a positive relationship between the covariate and the level of self-inhibition and a negative parameter estimate on a self-connection (negative effect of a covariate) indicates a negative relationship between the covariate and the level of self-inhibition.

For all other connections, positive parameters indicate that more excitatory/less inhibitory connectivity values are associated with higher PPBT scores (positive relationship), and negative parameter values indicate that more inhibitory/less excitatory connectivity values are associated with higher PPBT scores (negative relationship).

All non-zero parameter estimates had a posterior probability of 0.95 or greater with the exception of those with an asterisk. The posterior probabilities of the asterisked values are: STN→STR: 0.51, THAL→STN 0.5, R Cerebellum→M1
