# Supplementary material for: Motor network dynamic resting state fMRI connectivity of neurotypical children in regions affected by cerebral palsy
Source: Front Hum Neurosci. 2024 May 21;18:1339324. doi: 10.3389/fnhum.2024.1339324 (PMC11148452; doi:10.3389/fnhum.2024.1339324)
Supplement: Supplementary file 5 [file Table_5.pdf]

Supplementary Table S5a. Left Hemisphere main and covariate effects summary.

From	To	Main EC	Age	Motor skill	Comments on Covariate Effects
Left			↑↓	↑↓	
M1	M1	Inhibition			
	Striatum		↑ Excitation		With increased age, net EC from M1 to the striatum becomes more similar to that seen in classic direct and indirect pathway models.
	STN	Inhibition		↓ Inhibition	With increased motor skill, net EC from M1 to the STN becomes more similar to that seen in the classic indirect pathway model.
	GPi				
	Thalamus	Inhibition	↓ Inhibition		With increased age, net EC from M1 to the thalamus becomes more similar to that seen in the classic direct pathway model
	Cerebellum		↑ Excitation	↑ Excitation	With both age and motor skill increases, there is increased cerebellar involvement with M1, possibly related to cerebellar roles in automaticity.
Striatum	M1	Excitation			
	Striatum	Inhibition	↑ Inhibition	↑ Inhibition	Increased inhibition with both age and skill, suggesting increased striatal self-regulation
	STN	Inhibition		↓ Inhibition	With increased motor skill, net EC from the striatum to the STN becomes more similar to that seen in the classic indirect pathway model
	GPi	Excitation			
	Thalamus	Inhibition			
	Cerebellum	Excitation	↓ Excitation	↓ Excitation	With both increased age and motor skill, net EC to cerebellum lowers since excitation to cerebellum increases from M1 (see above for Left M1→contralateral cerebellum) it's possible that the influence to cerebellum switches to being more cortical.
STN	M1	Excitation			
	Striatum	Excitation			
	STN	Inhibition		↑ Inhibition	Increased inhibition with increased skill, suggesting increased STN self-regulation with skill
	GPi				
	Thalamus	Excitation			
	Cerebellum	Excitation			
GPi	M1	Excitation		↑ Excitation	Increases in GPi activity more logically cause decreases in M1 activity in classic models of the direct and indirect pathway. This effect is unexpected and may be reflective of the subjects being in a resting state, net effects within the models, or accounted for by influences from other nuclei (e.g., substantia nigra).
	Striatum				
	STN			↑ Excitation	Increases in excitation from GPi to the STN with motor skill may be reflective of the subjects being in a resting state, net effects within the indirect pathway, or accounted for by influences from other nuclei (e.g., substantia nigra).
	GPi	Inhibition			
	Thalamus	Inhibition			
	Cerebellum	Excitation			
Thalamus	M1	Excitation			
	Striatum	Excitation	↑ Excitation	↑ Excitation	The increase in excitation from the thalamus to the striatum with both age and motor skill may be a result of indirect excitation to the striatum via M1; the thalamus has a direct connection to M1.
	STN	Inhibition		↓ Inhibition	Increases in STN inhibition with thalamic activity associated with motor skill reflect a more similar pattern to classic models of the indirect pathway.
	GPi				
	Thalamus	Inhibition			
	Cerebellum	Excitation		↓ Excitation	Although direct anatomical connections from the thalamus to the contralateral cerebellum have not been noted, EC connections from thalamus to the contralateral cerebellum have been noted and this connection is further effected by thalamotomy in patients with essential tremor [13,53].
Cerebellum	M1	Excitation	↓ Excitation	↓ Excitation	
	Striatum	Inhibition			
	STN	Inhibition			
	GPi		↑ Excitation	↑ Excitation	The connection from contralateral cerebellum to GPi and its association with motor skill is supported by Milardi et al 2016[77] and Neumann et al 2015[78].
	Thalamus	Inhibition		↓ Inhibition	We show a decrease in inhibitory EC from the contralateral cerebellum to the thalamus associated with increased motor skill. Connections between the contralateral cerebellum and GPi are involved in the adaptive control of reaching behavior (Chen, 2006)[93].
	Cerebellum	Inhibition	↑ Inhibition		Increased inhibition with increased age, suggesting increased STN self-regulation with age

Supplementary Table S5b. Right Hemisphere main and covariate effects summary

From	To	Main EC	Age	Motor skill	Comments
Right			↑↓	↑↓	
M1	M1	Inhibition			
	Striatum	Inhibition		↓ Inhibition	The decrease in inhibition from M1 to striatum with motor skill appears more similar to the excitation from M1 to striatum seen in the classic direct and indirect path models.
	STN	Inhibition			
	GPi	Inhibition			
	Thalamus	Inhibition	↓ Inhibition	↓ Inhibition	With age, and motor skill, the effect of M1 on the thalamus is more similar to net effect seen in the classic direct pathway model.
	Cerebellum	Excitation		↑ Excitation	With motor skill increases, there is increased cerebellar involvement with M1, possibly related to cerebellar roles in automaticity.
Striatum	M1	Excitation			
	Striatum	Inhibition	↑ Inhibition		Increased inhibition with increased age, suggesting increased striatal self-regulation with age.
	STN	Inhibition	↑ Inhibition		Unlike the left hemisphere, increases in age are associated with the opposite effect expected for increased activity of the striatum on the STN within the classic indirect pathway model.
	GPi		↑ Excitation		With increased age, the EC from the striatum to GPi becomes more like the net effect expected in the classic indirect pathway model.
	Thalamus	Excitation			
	Cerebellum	Excitation			
STN	M1	Excitation			
	Striatum	Excitation			
	STN	Inhibition			
	GPi	Excitation		↑ Inhibition	With motor skill increases, EC from the STN to GPi become more inhibitory and thus more similar to the classic indirect pathway model.
	Thalamus	Excitation		↑ Inhibition	With motor skill increases, EC from the STN to the thalamus become less like the net effect expected in the classic indirect pathway model.
	Cerebellum	Excitation			
GPi	M1				
	Striatum		↑ Inhibition	↑ Excitation	The difference seen with age associated with an increase in inhibition and motor skill associated with an increase in excitation seems paradoxical but may reflect different trajectories or time frames of association of age and motor skill, respectively, with this connection.
	STN	Inhibition			
	GPi	Inhibition			
	Thalamus	Inhibition	↑ Inhibition	↓ Inhibition	The difference seen with age associated with an increase in inhibition and motor skill associated with a decrease in inhibition seems paradoxical but may reflect different trajectories or time frames of association of age and motor skill, respectively, with this connection.
	Cerebellum	Excitation		↑ Excitation	As there is no known direct connection between the GPi and the contralateral cerebellum, the increase in excitation with motor skill may reflect interconnectivity via the thalamus[104].
Thalamus	M1	Excitation	↓ Excitation	↑ Excitation	The thalamus excites M1 in classic models of the direct and indirect pathways. It is unclear why age and motor skill have opposite effects in this excitation, but it may reflect different trajectories or time frames of association of age and motor skill, respectively, with this connection.
	Striatum	Excitation			
	STN	Inhibition			
	GPi	Inhibition		↑ Inhibition	As motor skill increases, net EC (inhibition) from the thalamus to GPi increases, thus becoming more similar to the classic model of the indirect pathway.
	Thalamus	Inhibition		↓ Inhibition	This effect seems paradoxical, as increased self-inhibition with motor skill seems more advantageous and similar to other self-regulation trends.
	Cerebellum	Excitation			
Cerebellum	M1		↑ Inhibition		Increased role of cerebellum in automaticity in movement
	Striatum	Inhibition			
	STN	Inhibition	↑ Inhibition		There is a known anatomical pathway between STN and the contralateral cerebellum, however it is generally thought to run from the STN to the contralateral cerebellum [105].
	GPi	Inhibition	↓ Inhibition	↑ Inhibition	It is unclear why age and motor skill have opposite effects on inhibition from the contralateral cerebellum to the GPi, but it may reflect different trajectories or time frames of association of age and motor skill, respectively, with this connection. The connection from contralateral cerebellum to GPi is supported by Milardi et al 2016[77] and Neumann et al 2015[78]
	Thalamus	Inhibition			
	Cerebellum	Inhibition	↑ Inhibition	↑ Inhibition	Increased inhibition with increased age and motor skill, suggesting increased cerebellar self-regulation with age and motor skill.

Supplementary References

Pelzer EA, Melzer C, Timmermann L, von Cramon DY, Tittgemeyer M. Basal ganglia and cerebellar interconnectivity within the human thalamus. Brain Struct Funct. 2017;222(1):381-392. doi: 10.1007/s00429-016-1223-z.

Wang Z-M, Wei P-H, Shan Y, et al. Identifying and characterizing projections from the subthalamic nucleus to the cerebellum in humans. NeuroImage. 2020;210:116573. doi: 10.1016/j.neuroimage.2020.116573.
